# Reducing consumer pressure increases community dissimilarity leading to coral‐ or algal‐dominance on a coral reef

**DOI:** 10.1002/ecy.70507

**Published:** 2026-09-15

**Authors:** Kelly E. Speare, Thomas C. Adam, Allison Aplin, Noe Castañeda, Lauren N. Enright, Hannah E. Epstein, Jordan P. Gallagher, Adam Goodman, Olivia Isbell, Christian John, Mackenzie A. Kawahara, J. Grace Klinges, Mark C. Ladd, Jamie M. McDevitt‐Irwin, Rowan H. McLachlan, Nury Molina, Joseph R. Peters, Julianna J. Renzi, Andrew A. Shantz, Andrew R. Thurber, Alex D. Vompe, Rebecca Vega Thurber, Deron E. Burkepile

**Affiliations:** ^1^ Marine Science Institute University of California, Santa Barbara Santa Barbara California USA; ^2^ School of Geographical Sciences and Urban Planning Arizona State University Tempe Arizona USA; ^3^ Department of Biological Sciences Texas Tech University Lubbock Texas USA; ^4^ Department of Ecology, Evolution, and Marine Biology University of California, Santa Barbara Santa Barbara California USA; ^5^ School of Life Sciences University of Essex Colchester UK; ^6^ Department of Microbiology Oregon State University Corvallis Oregon USA; ^7^ Department of Biological Sciences Florida State University Tallahassee Florida USA; ^8^ Bren School of Environmental Science & Management University of California, Santa Barbara Santa Barbara California USA; ^9^ Island Conservation Santa Cruz California USA; ^10^ Population and Ecosystems Monitoring Division NOAA Southeast Fisheries Science Center Miami Florida USA; ^11^ School of Life Sciences University of Hawai'i at Mānoa Honolulu Hawai'i USA; ^12^ Oregon Institute of Marine Biology University of Oregon Charleston Oregon USA; ^13^ Cooperative Institute of Marine & Atmospheric Research University of Hawai'i at Mānoa Honolulu Hawai'i USA; ^14^ Department of Plant Pathology and Environmental Microbiology The Pennsylvania State University University Park Pennsylvania USA

**Keywords:** beta diversity, community assembly, community dispersion, corallivory, herbivory, nutrient enrichment, predation

## Abstract

In many ecosystems, anthropogenic influences are altering the strength of top‐down (e.g., herbivory and predation by consumers) and bottom‐up (e.g., nutrients) processes through the exploitation of consumers and increasing anthropogenic nutrient inputs. Altered top‐down and bottom‐up processes may significantly change ecological communities, resulting in shifts in the abundance or composition of primary producers, or shifts in ecosystem states. An often‐overlooked consequence of altered top‐down and bottom‐up process is how these forces shape community variability, especially following disturbances when alterations to top‐down and bottom‐up processes may have particularly strong effects during community reassembly. Here, we experimentally test the relative importance of top‐down and bottom‐up processes in determining community development after a simulated disturbance on a coral reef in Moorea, French Polynesia. We established eight 30‐m^2^ plots in which corals and macroalgae were removed to mimic the effects of a severe cyclone. Within each plot, we nested four consumer exclosures (~1.25 m^2^ each) with different size openings that allowed different size fishes access to the benthos, creating four levels of consumer pressure (Very Low, Low, Medium, High). Each plot was then assigned to either Ambient or Enriched nutrient conditions. Consumer pressure strongly affected community development, whereas nutrients had a weaker, but significant, effect. After 4 years, consumer pressure was inversely related to beta diversity, where plots with High consumer pressure had highly similar communities, and plots with reduced consumer pressure had dissimilar communities. This dissimilarity in community structure under reduced consumer pressure was the result of plots becoming rapidly colonized by either coral or macroalgae. By contrast, community development in plots with High consumer pressure was slow but predictable, with all plots slowly increasing in coral cover and remaining relatively free of macroalgae. Our results suggest that reduced top‐down pressure by consumers can result in alternate community paths that lead to either coral‐ or algal‐dominance under similar environmental conditions. We conclude that consumer pressure influences both the rate and predictability of community development in these ecosystems and that human stressors that alter consumer pressure may result in ecological surprises on coral reefs.

## INTRODUCTION

Two of the most pervasive impacts that humans have on the world's ecosystems are the overexploitation of animals and increases in nutrient availability. These two impacts can dramatically change the roles that consumers (top‐down processes) and nutrients (bottom‐up processes) have in driving community dynamics (Pace et al., 1999). For example, the loss of herbivores can result in increased biomass of primary producers and shifts in community composition, or even transitions to different ecosystem states (Andersen et al., 2009; Post, 2013; Scheffer et al., 2001). Changes in bottom‐up forcing, such as through the addition of nutrients, can also have profound effects on community dynamics by increasing primary production, altering producer abundance and diversity, and triggering shifts in ecosystem states (Adam et al., 2021; Menge, 2000; Scheffer et al., 2003). Importantly, the effect of consumers may depend on the availability of nutrients (Hillebrand et al., 2007), making it difficult to predict how anthropogenic alterations to top‐down and bottom‐up processes affect ecological communities. The compounding effects of altered consumer pressure and nutrient availability may result in ecological surprises where ecosystems transition to novel assemblages or ecosystem states (Filbee‐Dexter et al., 2017; Paine et al., 1998).

One effect of reduced consumer pressure or increased nutrient availability may be an increase in the variability of the dynamics of primary producers (Chen et al., 2022; Hautier et al., 2020). Herbivores can homogenize communities by consuming palatable taxa, increasing the relative abundance of taxa with higher tolerance to herbivory, and shrinking the species pool (Alberti et al., 2017; Anderson et al., 2011; Holmes & Webster, 2011; Socolar et al., 2016), which can result in low between‐site dissimilarity (i.e., beta diversity; Whittaker, 1972). When consumer pressure is reduced, such as through overexploitation of animals, the diversity of the species pool often increases, which can increase the importance of stochastic processes in determining community composition (Alberti et al., 2017; Chase et al., 2009). Thus, the loss of consumers can result in more dissimilar communities (i.e., higher beta diversity). Similarly, nutrient enrichment can increase community variability in plant communities (Chalcraft et al., 2008; Koerner et al., 2016). These effects of reduced consumer pressure or nutrient enrichment on beta diversity may be especially strong following disturbances where priority effects can determine community trajectories early in succession (Adam et al., 2022; Masterson et al., 2008). Although humans are altering bottom‐up and top‐down processes across many ecosystems, the consequences on community dissimilarity are not yet well understood (Socolar et al., 2016).

Coral reefs are subject to severe anthropogenic changes to top‐down and bottom‐up processes, driving reef degradation and regime shifts from coral‐ to macroalgal‐dominance (Elmhirst et al., 2009; Schmitt et al., 2019). Herbivores (e.g., fishes and sea urchins) are important determinants of community dynamics on coral reefs (Burkepile & Hay, 2006; McClanahan, 1997; Schmitt et al., 2019), and the role of herbivores may be especially important after disturbances because herbivores can reduce the abundance of macroalgae and facilitate the recovery of corals (Adam et al., 2011; Arnold et al., 2010). However, other work has questioned this strong and deterministic impact of herbivores on reef resilience after disturbances (Bruno et al., 2019; Cline & Allgeier, 2022) suggesting that this process is more stochastic than previously assumed. Increasing nutrient availability can also be an important determinant of coral reef community dynamics (Burkepile & Hay, 2006; Lapointe et al., 1997; Szmant, 2002) with excess nutrients typically increasing the abundance of macroalgae at the expense of coral abundance (Adam et al., 2021; Lapointe et al., 2021). However, an often‐overlooked aspect in the debate about the roles of top‐down and bottom‐up processes on coral reefs is how these forces shape community variability (but see McDevitt‐Irwin et al., 2023), especially following disturbances when alterations to top‐down and bottom‐up processes may have particularly strong effects during community reassembly.

Here, we experimentally test the relative importance of alterations to top‐down and bottom‐up processes in driving community development and variability after a simulated disturbance on a coral reef. We use a factorial experiment manipulating consumer pressure and nutrient enrichment for 4 years on a coral reef in Moorea, French Polynesia. Our work builds on foundational consumer—nutrient experiments by creating multiple levels of consumer exclusion (i.e., a gradient) to mimic how anthropogenic forces reduce, but generally do not eliminate, assemblages of consumers in nature. Our experiment consisted of plots (~1.25 m^2^ each) that manipulated access by consumers via different size openings that allowed different size fishes access to the benthos, creating four levels of consumer pressure (Very Low, Low, Medium, High). These four levels of consumer pressure were crossed with manipulations of nutrient availability with either ambient or nutrient‐enriched conditions. At the start of the experiment, we removed all live corals and fleshy macroalgae from the plots to mimic the effects of a severe cyclone. We quantified the abundance of benthic organisms (e.g., corals, macroalgae) for 4 years to assess how experimental treatments affected community development. We then tested the hypotheses that reduced consumer pressure and the addition of nutrients leads to increased community variability (i.e., higher beta diversity) and less predictable patterns of community development.

## METHODS

### Site description and experimental design

**FIGURE 1:**
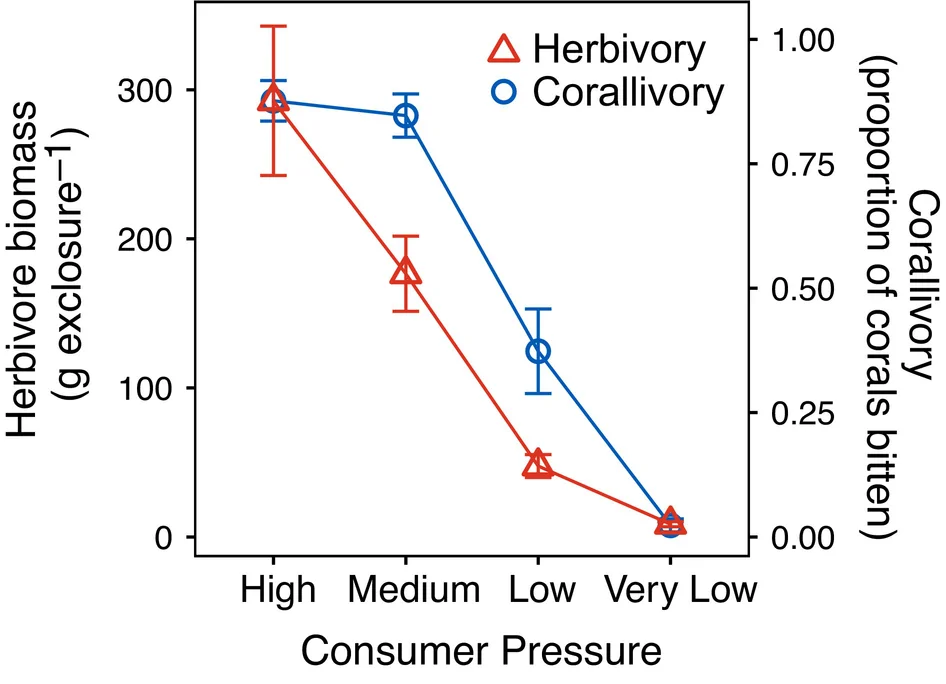
Metrics of herbivory (biomass of herbivores, in grams) and corallivory (proportion of corals bitten) across levels of consumer pressure. The probability of each coral having visible bite marks from corallivores is shown in blue, and the biomass of herbivores is shown in red. Error bars represent SEs.

**FIGURE 2:**
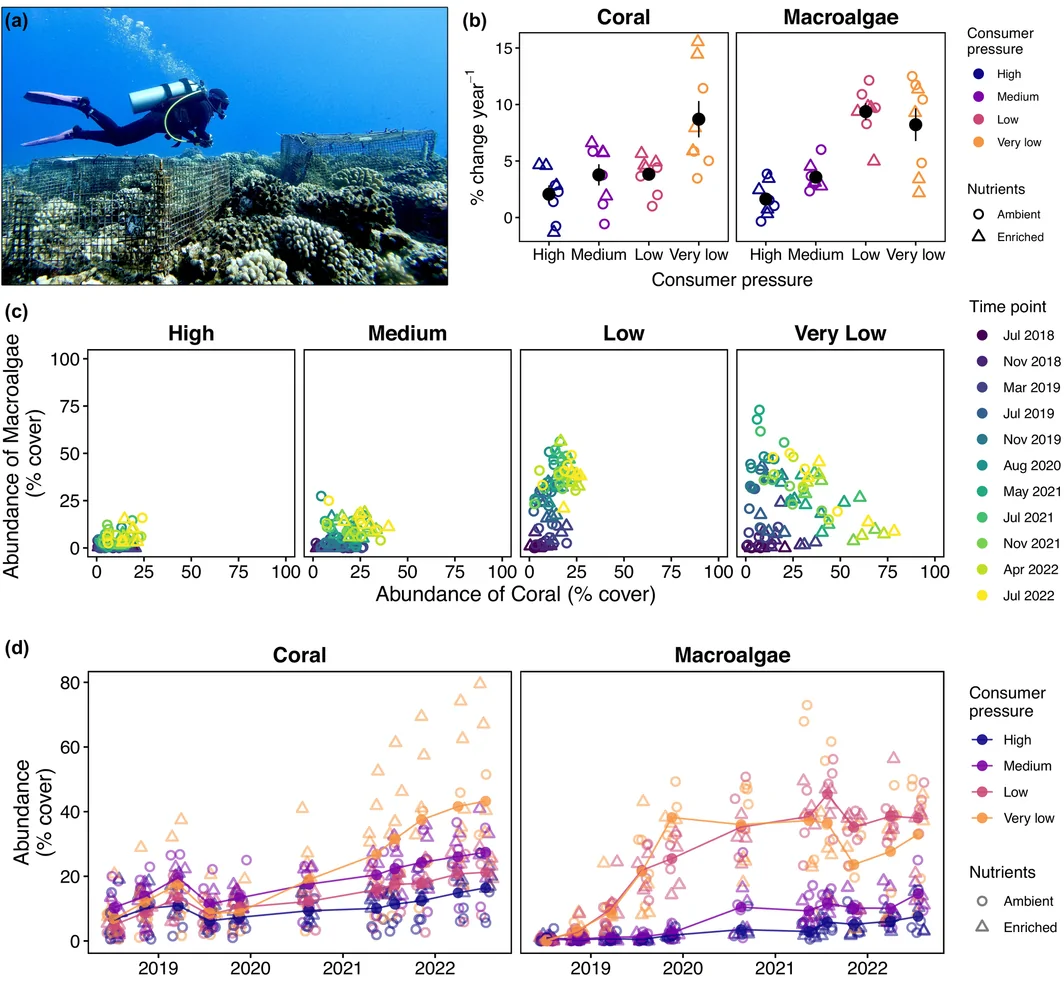
(a) Photo of a diver swimming over two of the exclosures in a plot. Each exclosure is approximately 1.12 m × 1.12 m. Photo by Allison Aplin. (b) Mean annual change in abundance of coral and macroalgae in experimental plots. Colored points represent replicate plots and black points are the mean of each consumer pressure treatment; error bars are SEs. Circles represent plots with ambient nutrients and triangles represent plots with enriched nutrients. (c) Biplot of the abundance of coral and macroalgae where panels are different levels of consumer pressure. Points are colored by time point with blue time points representing earlier in the experiment and yellow time points representing later in the experiment. Circles represent plots with ambient nutrients and triangles represent plots with enriched nutrients. (d) The abundance of corals and algae throughout the experiment. Open points are raw data and closed points are means by consumer pressure level. Circles represent plots with ambient nutrients and triangles represent plots with enriched nutrients.

In June 2018, we established a factorial experiment manipulating consumer pressure and nutrient availability in areas that mimicked disturbance from a cyclone. At 12 m depth on the north shore forereef of Moorea, French Polynesia (17.47° S, 149.82° W), we established eight approximately 30 m^2^ plots. Within each plot, we then nested four different consumer exclosures (~1.25 m^2^ each) with different size openings that allowed different size fishes (herbivores and corallivores, hereafter referred to as “consumers”) access to the benthos (Figures 1 and 2a). The exclosure frames consisted of 0.5 cm stainless steel all‐thread drilled into the reef matrix and epoxied into place. These frames were then wrapped with plastic‐coated, galvanized wire to create the following levels of consumer pressure: (1) Very Low (2.5 cm × 2.5 cm openings); (2) Low (5 cm × 5 cm openings); (3) Medium (7.5 cm × 7.5 cm openings); (4) High (4 sides of 2.5 cm × 2.5 cm openings but no top). Others have used a similar design to create a gradient of consumer pressure to mimic the effects of different levels of fishing (Holbrook et al., 2016; Schmitt et al., 2019). We included sides, but not tops, on the High consumer pressure treatment to control for potential artifacts on water flow, although we have shown these are minimal (Zaneveld et al., 2016). Exclosures were scrubbed every 12–16 weeks to remove fouling organisms. We quantified the biomass of fishes in our exclosures through timed in situ counts of fish (Figure 1). Corallivores are low in abundance relative to other fishes, making it difficult to quantify their abundance using surveys. Therefore, we quantified the proportion of corals with visible bite marks as a metric of corallivory (Figure 1). The full methods are described in Appendix S1, and the biomass of fishes in our treatments is shown in Appendix S1: Figures S2 and S3. Herbivory on the forereef is almost exclusively from fishes as grazing sea urchins are not abundant, but in the rare instances when urchins colonized our exclosures, we removed them (<10 urchins removed over the duration of the 4‐year experiment).

Within the exclosures, we simulated the effects of a cyclone. To do so, we removed all branching corals (mostly *Acropora* spp. and *Pocillopora* spp.) and transplanted them to other areas of the reef away from the experiment. The removal of branching corals simulated a pulse disturbance such as the cyclone that reduced coral cover to <5% on the forereefs of Moorea in 2010 (Adam et al., 2011). Remaining patches of encrusting corals (mostly *Montipora* spp.) and mounding corals (mostly *Porites* spp.) and macroalgae (which were rare) were scrubbed with wire brushes to mimic the scouring from sediments that occurs during a large cyclone. Thus, after coral and macroalgae removal, mean coral cover in the plots was approximately 7% and macroalgae was approximately 0.5%.

Each plot (with four nested consumer exclosures) was then assigned to either Ambient or Enriched nutrient conditions. Thus, each combination of consumer pressure (Very Low, Low, Medium, High) and nutrients (Ambient or Enriched) had *n* = 4 for replication (see Appendix S1: Figure S1). For the enrichment, we placed 175 g of Osmocote (19–6–12, N–P–K) slow‐release garden fertilizer into 5 cm diameter polyvinyl chloride (PVC) tubes with 10, 1‐cm holes drilled into them. These tubes were wrapped in fine plastic mesh to retain the fertilizer. This method is similar to our previous work (e.g., Burkepile et al., 2020; Zaneveld et al., 2016). PVC enrichment tubes were attached to the corners of each exclosure and onto a piece of stainless steel all‐thread in the center of each plot (five enrichment tubes per exclosure). We replaced enrichment tubes every 12–16 weeks except for two periods during the COVID‐19 pandemic when travel to Moorea was not possible and enrichment tubes were deployed for longer than usual before replacement (deployed from 30 January 2020 to 31 August 2020, and from 31 August 2020 to 8 February 2021). We analyzed water samples from the experimental plots to evaluate the effect of nutrient enrichment. We showed that the enrichment treatment effectively enriched nutrients throughout the experiment, but that the effect of the enrichment decreased with time since replacement of enrichment tubes. This suggests that the enrichment may have been negligible by the end of each deployment of the enrichment tubes, and during the two periods during the pandemic when tubes were deployed longer than usual. Full methods and results are described in Appendix S1.

### Quantifying the abundance of benthic taxa

At three time points each year (April, July/August, November—except for two time points missed due to the COVID‐19 pandemic, April 2020 and November 2020), we quantified benthic cover in each exclosure via point contacts on orthorectified photomosaics. Using Olympus TG cameras, we took approximately 64 individual photographs of each exclosure and then generated photomosaics by stitching photographs together using Agisoft Metashape software (Agisoft Metashape Professional Version 17.0). These photomosaics generate high‐resolution imagery that allows identification of benthic space holders to the lowest taxonomic level possible (genus for most corals and macroalgae). It was not possible to reliably distinguish among filamentous turf algae, bare space, and very small encrusting algae (including small crustose coralline algae and other encrusting algae) from photographs; therefore, these taxa were categorized as one group. We used these data to generate percent cover estimates for each exclosure at each time point. Percent cover of benthic space holders was estimated via 225 random points per exclosure using CoralNet software (Beijbom et al., 2015).

### Statistical analyses

#### Effects of consumer pressure and nutrient enrichment on benthic communities

To evaluate how consumer pressure and nutrient enrichment influenced the rate of community development, we calculated the rate of increase of corals and macroalgae, the two most abundant groups of benthic organisms in our plots, from July 2018 to July 2022. Rates were calculated as the absolute annual rate of change, which was the difference in abundance from 2018 to 2022, divided by 4 years. Then, we fit two mixed‐effects models to evaluate how consumer pressure, nutrient enrichment, and their interaction influenced the rate of increase of corals or macroalgae. Models had a random effect of Block. Models were constructed using the *lme4* package (Bates et al., 2015), and χ^2^ tests were performed with Type II Sums of Squares using the *anova* function from the *car* package (Fox & Weisberg, 2018). Model residual diagnostics were evaluated using the *DHARMa* package (Hartig, 2024). Statistical analyses and data visualization were performed in R version 4.3.

To assess whether consumer pressure, nutrients, or their interaction had a significant effect on the composition and abundance of taxa in communities at the start of the experiment (July 2018) and after 4 years (July 2022), we used a permutational analysis of variance (PERMANOVA) with the *adonis2* function in the *vegan* package (Oksanen et al., 2022). Tests were done on each time point separately. If differences in communities across treatments were present, we then computed similarity percentages (SIMPER) using *simper* (Clarke, 1993) in *vegan* to contrast communities in the consumer pressure and nutrient treatments. Nonmetric multidimensional scaling (NMDS) was used to visualize differences in communities in experimental exclosures, and distance matrices were computed using the Bray–Curtis dissimilarity index applied to untransformed abundance data.

#### Effects of consumer pressure and nutrient enrichment on beta diversity

We then quantified how consumer pressure and nutrient enrichment influenced multivariate group dispersion (hereafter referred to as “beta dispersion”), a measure of beta diversity (Anderson et al., 2006), by conducting an analysis of multivariate homogeneity of group dispersions (PERMDISP2) using *betadisper* in the *vegan* package (Anderson, 2006; Anderson et al., 2006; Oksanen et al., 2022). For all analyses, beta dispersion was calculated as the average distance of group members to the group spatial median based on Bray–Curtis dissimilarity matrices. We used the Bray–Curtis dissimilarity index to compute dissimilarity matrices because we were most interested in changes in relative abundance of taxa. Testing for multivariate homogeneity of dispersions across two factors, such as our experimental design, can be problematic if there is an interaction between the two main effects on the location of group spatial medians because differences in the locations of group spatial medians can influence beta dispersion. We describe how we evaluated and ruled out this possibility in Appendix S1 (see Additional Methods, and Additional Results in Tables S2–S13).

To evaluate how temporal patterns of beta dispersion related to the consumer pressure and nutrient treatments, we first calculated beta dispersion for all unique treatment combinations (8 treatments) for each experimental time point. To ensure that beta dispersion did not differ among treatments prior to our experimental manipulation, we tested for treatment effects on beta dispersion at the start of the experiment (July 2018) using *anova*. We then used a linear mixed‐effects model with beta dispersion as the response variable. The full model included individual effects of consumer pressure, nutrients, and days since the start of the experiment (hereafter referred to as “time”), and all two‐way interactions. The three‐way interaction was initially included in the model but it was not significant, so it was removed (three‐way interaction: χ^2^ = 0.839, df = 3, *p* = 0.840). Plot identity was included as a random effect to account for the repeated measures design. Models were constructed using the *lme4* package (Bates et al., 2015), and χ^2^ tests were performed with Type II Sums of Squares using the *anova* function from the *car* package (Fox & Weisberg, 2018). Model residual diagnostics were evaluated using the *DHARMa* package (Hartig, 2024). We used *emtrends* to compute post hoc contrasts of slopes for different levels of fixed effects.

#### Relationships between the availability of colonizable space and species rank abundances on beta diversity

At the start of the experiment, colonizable space (which we defined as the abundance of turf algae, bare space, and encrusting algae) was abundant and other benthic taxa colonized this available space throughout the experiment. We hypothesized that community dissimilarity resulted from frequent reordering of species when colonizable space was abundant, and less reordering when colonizable space was scarce. These patterns could arise because once space is occupied by a particular taxon (e.g., corals, algae) it becomes more difficult for other taxa to increase in abundance. We evaluated relationships between time, the availability of colonizable space, and the mean rank shift (MRS) metric which measures the reordering of taxa (Avolio et al., 2015; Collins et al., 2008). MRS is a relative measure of changes in species rank abundance between pairs of time points (Collins et al., 2008) where higher MRS indicates more shifts in rank abundance relative to lower MRS. For example, a MRS of two indicates that on average taxa shifted by two positions between pairs of time points. For each exclosure, we calculated MRS of taxa between each pair of consecutive time points using the *codyn* package (Hallett et al., 2016).

Because the abundance of colonizable space was highly correlated with time, and the range of colonizable space differed across levels of consumer pressure, it was necessary to evaluate relationships between time and MRS or colonizable space in separate models with the same structure. We fit three separate mixed‐effects models. First, we evaluated how the abundance of colonizable space related to time, consumer pressure, nutrients and their interactions. Second, we evaluated how MRS related to time, consumer pressure, nutrients, and their interactions. Third, we evaluated how beta dispersion related to MRS, time, consumer pressure, nutrients, and their interactions. In all models, the three‐way interactions were not significant, so they were removed. Models included plot identity as a random effect to account for the repeated measures design. Models were constructed using the *lme4* package (Bates et al., 2015) and χ^2^ tests were performed with Type II Sums of Squares using *anova* from the *car* package (Fox & Weisberg, 2018). Model residual diagnostics were evaluated using the *DHARMa* package (Hartig, 2024). We used *emtrends* to compute post hoc contrast of slopes for different levels of fixed effects.

## RESULTS

### Benthic communities are strongly influenced by consumer pressure and weakly influenced by nutrient enrichment

Over the 4‐year experiment, consumer pressure had a strong negative effect on the rate of increase of corals, where coral cover increased at a higher rate at lower consumer pressure (mixed‐effects model, χ^2^ = 32.06, df = 3, *p* < 0.0001, Figure 2b). Increases in coral cover were driven primarily by recruitment of new corals (K.E. Speare, personal observations). Nutrient enrichment had a weaker but positive effect on corals, where coral cover increased at a higher rate in the enriched treatment (mixed‐effects model, χ^2^ = 8.75, df = 1, *p* = 0.003). There was not a significant interaction between consumer pressure and nutrients on the rate of increase of corals (mixed‐effects model, χ^2^ = 1.56, df = 3, *p* = 0.668). Similarly, consumer pressure had a strong negative effect on the rate of increase of macroalgae (mixed‐effects model, χ^2^ = 55.94, df = 3, *p* < 0.0001, Figure 2b) where macroalgae increased at a higher rate at lower consumer pressure. Nutrient enrichment did not have a significant effect on the rate of increase of macroalgae (mixed‐effects model, χ^2^ = 2.41, df = 1, *p* = 0.120). There was not a significant interaction between consumer pressure and nutrients on the rate of increase of macroalgae (χ^2^ = 2.57, df = 3, *p* = 0.462). At High consumer pressure, community development was relatively consistent among replicates, with plots increasing in coral cover slowly (2.5% ± 0.8% mean ± SE increase in coral cover per year, Figure 2c,d) and remaining relatively free of macroalgae (1.6% ± 0.5%, mean ± SE increase in macroalgae per year, Figure 2c,d). By contrast, under Very Low consumer pressure, corals (9.0% ± 1.6, mean ± SE % increase per year) and/or macroalgae (8.2% ± 1.4% increase per year) increased in abundance rapidly, with several plots exceeding 50% cover of coral or macroalgae within 4 years (Figure 2d). The abundance of coral and macroalgae varied both across and within levels of the consumer pressure treatment, where some plots had abundant macroalgae with few corals, some plots had high coral cover and little macroalgae, and some plots had abundant coral and macroalgae.

**FIGURE 3:**
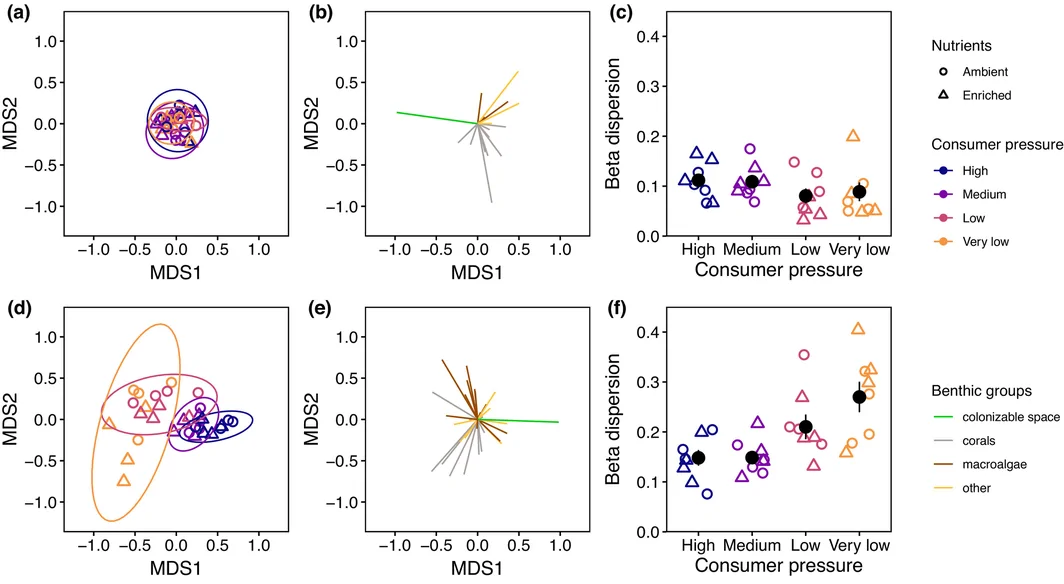
Nonmetric multidimensional scaling (NMDS) plots to visualize differences in community composition between experimental plots (a) at the start of the experiment and (d) after 4 years. In (a, d) point colors represent different levels of consumer pressure and shapes represent different levels of nutrient enrichment. Experimental plots were grouped by level of consumer pressure and beta dispersion of the group was calculated for each point. Ellipses are 95% CIs. Taxon vectors that correspond to (a, d) are shown in (b, e). Vectors are shown for each taxon are colored by benthic group such that multiple lines of the same color represent different taxa in that group (e.g., multiple species of corals represented by multiple brown lines). See Appendix S1: Table S1 for list of taxa in each benthic group. Beta dispersion (i.e., a metric of beta diversity) values are shown for each replicate plot (c) at the start of the experiment and (f) after 4 years, where colored points represent replicate plots and black points are the mean of each consumer pressure treatment; error bars are SEs.

At the start of the experiment, community composition in experimental exclosures did not differ by consumer pressure treatment (PERMANOVA, *F*_(3,24)_ = 0.63, *p* = 0.781), nutrient treatment (PERMANOVA, *F*_(1,24)_ = 0.04, *p* = 0.958), or their interaction (*F*_(3,24)_ = 1.13, *p* = 0.340, Figure 3a,b). However, after 4 years, communities differed significantly by consumer pressure treatment (PERMANOVA, *F*_(3,24)_ = 10.57, *p* = 0.001), and nutrient treatment (PERMANOVA, *F*_(1,24)_ = 3.210, *p* = 0.018), but not their interaction (PERMANOVA, *F*_(3,24)_ = 1.10, *p* = 0.376, Figure 3d,e). Overall, consumers explained substantially more variation in community composition (PERMANOVA, *R* ^2^ = 0.51) than nutrients (PERMANOVA, *R* ^2^ = 0.05). Plots in the High versus Very Low consumer pressure treatments had the greatest difference in communities. Eighty percent of the differences in communities among these treatments were driven by differences in the abundance of six taxa/functional groups. Five of these groups, two coral (*Montipora* spp. and *Acropora* spp.) and three macroalgal (foliose *Lobophora* spp., *Turbinaria ornata*, and *Halimeda* spp.) taxa, were most abundant under Very Low consumer pressure, while the sixth group (turf, bare space, and encrusting algae) was most abundant under High consumer pressure (SIMPER, see Appendix S1: Tables S15–S20 for full list of taxa and comparisons of all treatments). Communities also differed in the ambient versus. enriched nutrient treatments at the end of the experiment. Plots that were enriched with nutrients tended to have high abundance of corals (*Montipora* spp., *Pocillopora* spp., and *Acropora* spp.), whereas plots with ambient nutrients tended to have more macroalgae (foliose *Lobophora* spp., *Halimeda* spp., *T. ornata*). The abundance of these coral and macroalgal taxa, in addition to turf, bare space, and encrusting algae, accounted for 80% of the difference in communities between enriched and ambient plots at the end of the experiment (SIMPER, see Appendix S1: Table S20 for full list of taxa).

### Reduced consumer pressure increases beta diversity through time

**FIGURE 4:**
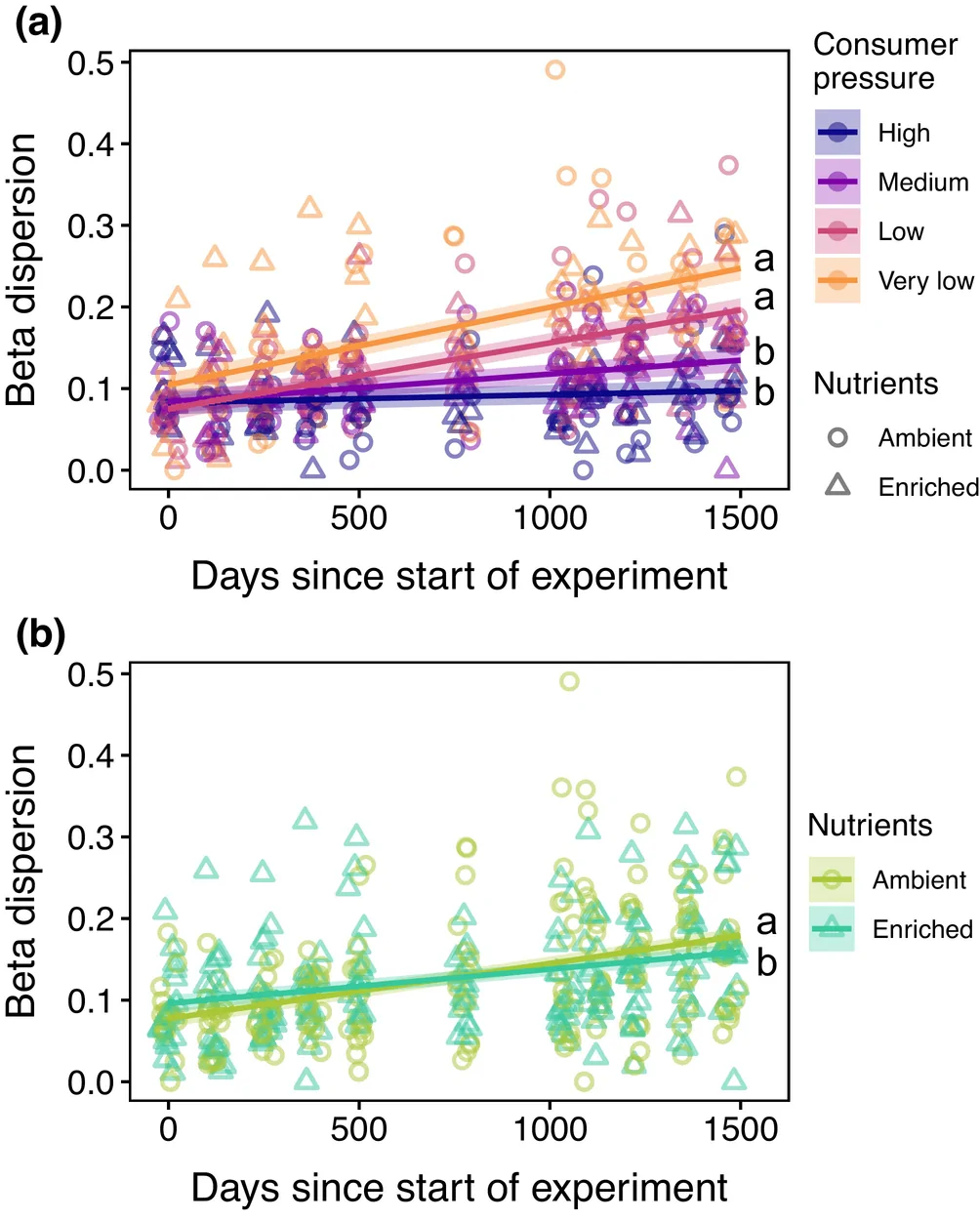
Temporal patterns of community dissimilarity, measured as beta dispersion, throughout the 4‐year experiment. Points represent the beta dispersion value for each replicate. Beta dispersion values were calculated for eight treatment combinations (e.g., High consumer pressure, Ambient nutrients) reflecting the factorial experimental design. Colors represent (a) levels of the consumer pressure treatment or (b) levels of the nutrient enrichment treatment. In both panels, points are the data from each plot and shapes represent levels of nutrient enrichment. Lines and shaded regions are the predicted relationships and SEs from the mixed‐effects models. Within a panel, letters represent Tukey's post hoc comparisons of slopes such that lines that do not share letters have significantly different slopes (*p* < 0.05).

To test the hypothesis that reduced consumer pressure and nutrient enrichment increase beta diversity, we quantified beta dispersion across treatments and time. At the beginning of the experiment, beta dispersion did not differ significantly based on consumer pressure (ANOVA, *F*_(3,28)_ = 1.08, *p* = 0.375, Figure 3a,c) or nutrients (ANOVA, *F*_(1,30)_ = 0.50, *p* = 0.486). When we tested how beta dispersion varied through time, there was a strong effect of time, where beta dispersion increased significantly over 4 years (mixed‐effects model, χ^2^ = 87.90, df = 1, *p* < 0.001). There was also a strong effect of consumer pressure, where beta dispersion increased as consumer pressure decreased (mixed‐effects model, χ^2^ = 31.32, df = 3, *p* < 0.001, Figure 4a), and a significant interaction between consumer pressure and time, where the effect of consumer pressure became stronger through time (mixed‐effects model, χ^2^ = 34.61, df = 3, *p* < 0.001, Figure 4a). There was not a significant main effect of nutrients (mixed‐effects model, χ^2^ = 0.003, df = 1, *p* = 0.96), but there was a significant interaction between nutrients and time, where beta dispersion increased at a higher rate in the ambient compared to enriched treatment (mixed‐effects model, χ^2^ = 4.80, df = 1, *p* = 0.028, Figure 4b). There was not a significant interaction between nutrients and consumer pressure (mixed‐effects model, χ^2^ = 1.00, df = 3, *p* = 0.802).

### Beta diversity increases as colonizable space and mean rank shifts decline under reduced consumer pressure

**FIGURE 5:**
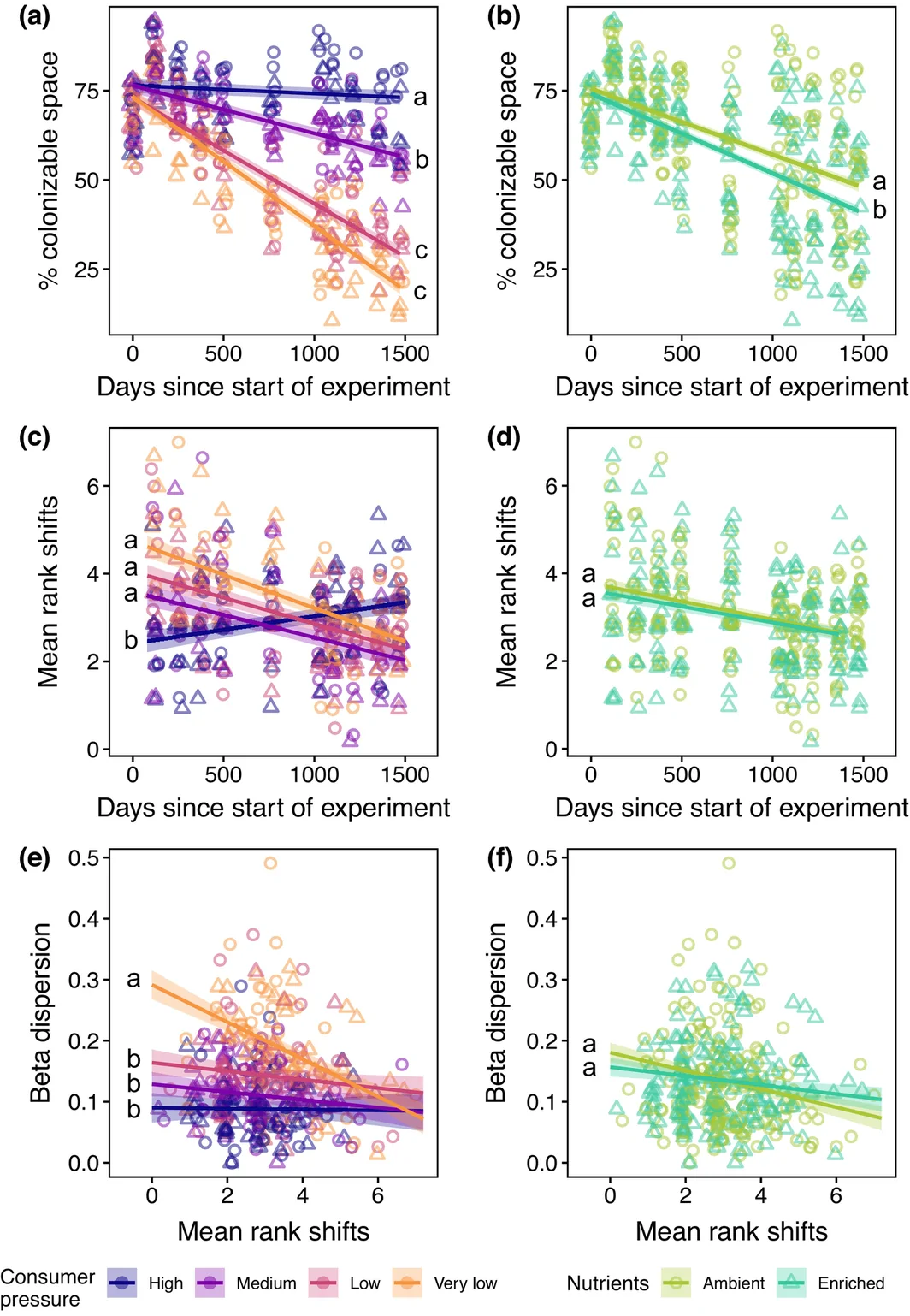
(a, b) The abundance of colonizable space plotted against time. (c, d) Mean rank shift (MRS) of taxa plotted against time. In (a, c, e) colors represent different levels of consumer pressure, and in (b, d, f) colors represent different levels of nutrients. In all panels, points are the raw data and shapes represent levels of nutrient enrichment. Lines and shaded regions are the predicted relationships and SEs from the mixed‐effects models. Within a panel, letters represent Tukey's post hoc comparisons of slopes such that lines that do not share letters have significantly different slopes (*p* < 0.05).

We hypothesized that benthic space would be colonized at different rates depending on consumer pressure and nutrients and that the availability of space would affect reordering of taxa abundances (i.e., MRSs). The abundance of colonizable space declined through time at different rates depending on treatment (Figure 5a,b) with significant effects of consumer pressure (mixed‐effects model, χ^2^ = 213.02, df = 3, *p* < 0.0001), nutrients (mixed‐effects model, χ^2^ = 7.15 df = 1, *p* = 0.007), and time (mixed‐effects model, χ^2^ = 507.87, df = 1, *p* < 0.0001) on colonizable space. There was also a significant interaction between consumer pressure and time on the abundance of colonizable space (mixed‐effects model, χ^2^ = 218.48, df = 3, *p* < 0.0001), where the abundance of colonizable space decreased significantly more quickly at the Low and Very Low levels of consumer pressure compared to Medium and High consumer pressure (Tukey's post hoc test, *p* < 0.05, Figure 5a). Similarly, there was a significant interaction between nutrients and time on the abundance of colonizable space (mixed‐effects model, χ^2^ = 4.57, df = 1, *p* = 0.033) where the abundance of colonizable space decreased more quickly in the enriched treatment compared to the ambient treatment (Tukey's post hoc test, *p* < 0.05, Figure 5b). The interaction between consumer pressure and nutrients was not significant (mixed‐effects model, χ^2^ = 1.61, df = 3, *p* = 0.658).

Patterns of MRSs of benthic taxa differed across experimental treatments and through time (Figure 5c,d). There were significant effects of consumer pressure (mixed‐effects model, χ^2^ = 11.98, df = 3, *p* = 0.007, Figure 5c) and time (mixed‐ effects model, χ^2^ = 38.62, df = 1, *p* < 0.001), but not of nutrient enrichment (mixed‐effects model, χ^2^ = 0.28, df = 1, *p* = 0.597, Figure 5d). There was also a significant interaction between consumer pressure and time on the number of MRS (mixed‐effects model, χ^2^ = 43.59, df = 3, *p* < 0.0001, Figure 5c). MRS decreased through time in the Medium, Low, and Very Low consumer pressure treatments, but not in the High consumer pressure treatment. There was not a significant interaction between nutrients and time (mixed‐effects model, χ^2^ = 0.15, df = 1, *p* = 0.703), or nutrients and consumer pressure (mixed‐effects model, χ^2^ = 1.71, df = 3, *p* = 0.635) on MRS.

We hypothesized that beta dispersion (i.e., beta diversity) increases as the reordering of species abundances (i.e., MRS) declines. The relationship between beta dispersion and MRS differed depending on consumer pressure but not nutrients (Figure 5e,f). There was a significant effect of MRS on beta dispersion (mixed‐effects model, χ^2^ = 16.23, df = 1, *p* < 0.0001) where beta dispersion was high when MRS was low. There was also a significant effect of consumer pressure on beta dispersion (mixed‐effects model, χ^2^ = 40.64, df = 3, *p* < 0.0001) where beta dispersion was higher at lower levels of consumer pressure. There was also a significant interaction between MRS and consumer pressure (mixed‐effects model, χ^2^ = 14.75, df = 3, *p* = 0.002) where there was a stronger relationship between MRS and beta dispersion at Very Low consumer pressure compared to all other levels of consumer pressure (Tukey's post hoc test, *p* < 0.05, Figure 5e). There was not a significant interaction between MRS and nutrient enrichment (mixed‐effects model, χ^2^ = 1.56, df = 1, *p* = 0.211), or consumer pressure and nutrient enrichment (mixed‐effects model, χ^2^ = 0.76, df = 3, *p* = 0.86).

## DISCUSSION

Our experiment shows that two anthropogenic stressors, reduction in consumer pressure and nutrient enrichment, have different effects on community development and variability following a disturbance on a coral reef. Increasing nutrient availability decreased community variability regardless of the level of consumer pressure. By contrast, gradually reducing consumer pressure led to increasingly divergent and unpredictable community development. Communities under the lowest consumer pressure were highly variable across space, with some plots dominated by macroalgae and others dominated by corals. Yet, the highest levels of consumer pressure resulted in the most predictable communities where macroalgae never became abundant but corals increased at a slower rate. As communities developed after the disturbance, reductions in consumer pressure led to less frequent changes in rank abundance of taxa (MRSs) as colonizable space decreased. Thus, the removal of consumers likely allowed for early colonizing species to become dominant, through priority effects (Fukami, 2015), resulting in differential community trajectories across replicates within treatments. Our experimental evidence shows that by reducing consumer pressure, overfishing can result in increasingly unpredictable patterns of community development on coral reefs.

Reducing consumer pressure had strong and rapid effects on community variability, with beta dispersion increasing rapidly at the lowest levels of consumer pressure (Figures 3d,f and 4a). This result is similar to other experiments excluding large vertebrate consumers that show increased variability in community composition under reduced consumer pressure (Alberti et al., 2017; Chase et al., 2009; McDevitt‐Irwin et al., 2023). Theory predicts that consumers favor deterministic processes during community assembly by targeting palatable taxa or taxa with particular nutritional value. This leads to a reduced pool of species that either tolerate or avoid consumers, resulting in predictable community development and low community variability when consumers are present (Chase et al., 2009). By contrast, increased community variability can result when consumer pressure is reduced because stochastic processes become more important in community assembly. One important process likely driving increased variability at lower levels of consumer pressure is priority effects, where the sequence of species arrival determines the community state through either niche preemption (i.e., taxa that arrive early use up existing resources) or niche modification (i.e., taxa that arrive early modify the environment) (Fukami, 2015). We previously showed that there were strong priority effects in this system where either corals or macroalgae strongly inhibited colonization by the other when they colonized first (Adam et al., 2022). Therefore, the loss of consumers on coral reefs may increase the role of priority effects as similarly shown in aquatic ecosystems (Louette & De Meester, 2007) because low consumer pressure may increase the success of initial colonizing taxa, resulting in eventual dominance by the taxa that arrive early. Thus, consumers may act as a homogenizing force by removing a subset of colonizing species and limiting the impact of priority effects on community development.

The idea that priority effects contributed to increased community variability is supported by the contrasting patterns of reordering of species rank abundances (i.e., MRSs) at different levels of consumer pressure. In the three treatments where consumer pressure was reduced (Medium, Low, and Very Low consumer pressure), MRSs were highest early in the experiment when colonizable space was abundant (Figure 5a,c). Taxa quickly colonized available space, resulting in frequent reordering of species abundances early in the experiment (i.e., high MRS, Figure 5a,c). However, as the abundance of colonizable space declined, the number of changes in rank abundances also declined sharply (i.e., low MRS later in the experiment, Figure 5c), in contrast to High consumer pressure where MRS increased over time. This suggests that communities under lower consumer pressure became locked into a particular community state as colonizable space became scarce, while communities in High consumer pressure undergo more community changes over time. Additionally, colonizable space declined at similar rates in the Low and Very Low consumer pressure treatments (Figure 5a), but there was a stronger relationship between MRS and beta dispersion at Very Low consumer pressure. This suggests that consumer pressure, in addition to the effects of space availability, modulates how species reordering translates to dissimilar community trajectories.

**FIGURE 6:**
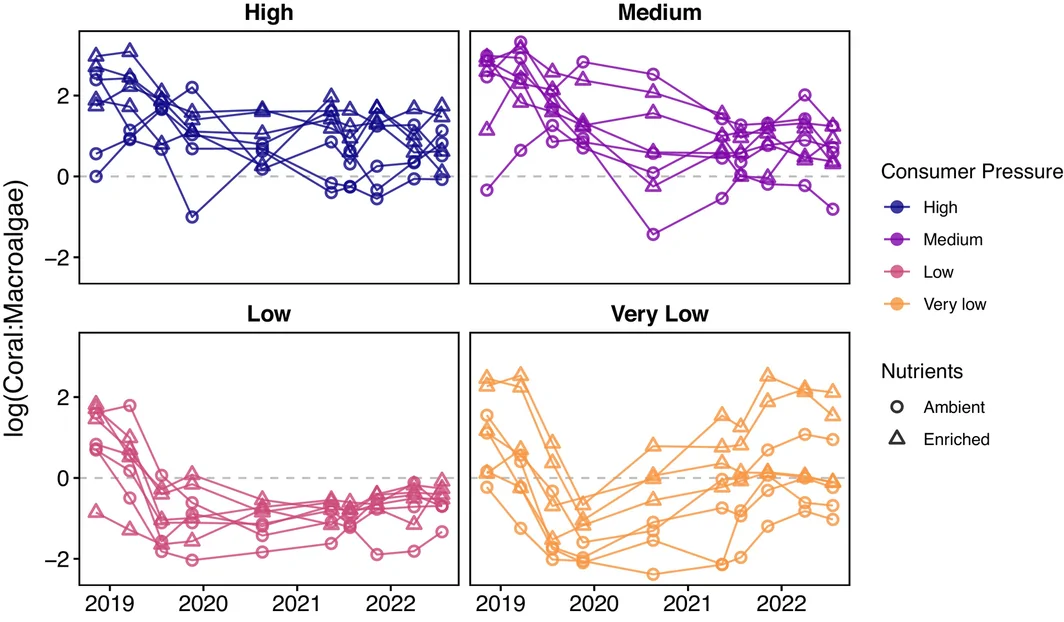
The log ratio of corals to macroalgae in each plot versus time. Colors represent different levels of consumer pressure and shapes represent different levels of nutrients. Lines connect points from the same experimental plot through time. The dotted line at zero represents equal abundance of corals and macroalgae. Points above the dotted line have more coral than macroalgae, while points below the dotted line have more macroalgae than coral.

In contrast to the effects of losing consumers, the addition of nutrients decreased community variability in our experiment. However, the effect of nutrients was weaker than the effect of consumers. The addition of nutrients can have positive (Chalcraft et al., 2008; Chase, 2010; Houseman et al., 2008; Koerner et al., 2016) or negative (Chalcraft et al., 2008; Molina et al., 2021) effects on community variability. Nutrient enrichment may increase community variability by increasing productivity and stochastic processes that lead to dissimilar community development (Chase, 2010). By contrast, nutrients may decrease community variability by decreasing the niche space (Harpole & Tilman, 2007) and the number of species that can exist under enriched conditions, or by altering competitive outcomes if certain species are better able to use nutrients to increase biomass (e.g., niche preemption) (Chase, 2010; Cook et al., 2018; Molina et al., 2021). For example, in terrestrial grasslands, the addition of nutrients often results in reduced species richness because species that can persist in low nutrient conditions get outcompeted by those that require more nutrients (Harpole & Tilman, 2007; Muehleisen et al., 2023). In our experiment, plots with elevated nutrients tended to have more corals than macroalgae (Figures 3d,e and 6), suggesting that nutrients benefited corals through increased growth or survival, by giving corals a competitive edge over macroalgae, or by suppressing macroalgae via some other mechanism (Rasher et al., 2012). This result is surprising given that corals and algae compete for space on coral reefs, and nutrient enrichment has been implicated in the decline of corals and increase in the abundance of macroalgae (Adam et al., 2021; Lapointe et al., 2021). However, some field and lab studies have shown that nutrients can benefit corals by increasing the density of algal symbionts, which can benefit corals by increasing photosynthesis and colony growth (Becker et al., 2021; Ezzat et al., 2016; Shantz & Burkepile, 2014). The addition of both nitrogen and phosphorus, which was the case in our experiment (Appendix S1: Figure S4), may be particularly beneficial to corals (Ezzat et al., 2015), and the level of enrichment in our experiment was modest which may also benefit corals rather than harm them (Becker et al., 2021). Rapid increases in the abundance of corals, mediated by nutrient enrichment, resulted in increased community similarity (i.e., decreased beta diversity) under nutrient enrichment.

The effect of nutrients was relatively weak in our experiment, but it is possible that the true effect of nutrients is stronger than our results suggest. Our enrichment treatment doubled nitrate + nitrite from 0.2 to 0.4 μM; thus, the level of enrichment in our experiment was low. Water samples showed that water nutrient concentrations declined throughout the deployment of the enrichment tubes (Appendix S1: Figure S4) and that water from the enriched plot was not significantly elevated in Nitrate + Nitrite or Phosphorus relative to water from ambient plots by the time diffusers were exchanged (after ~150 days of deployment). This suggests that our nutrient enrichment was modest, and exchanging the enrichment tubes more frequently might have resulted in stronger effects of nutrients.

Herbivores are thought to facilitate corals by controlling algae and promoting coral recruitment, and their effects may be especially strong during recovery after disturbances (Adam et al., 2011). Surprisingly, we found that consumers slowed the rate of increase of corals after disturbance in our experiment. On average, coral cover increased at a higher rate in the Very Low consumer pressure treatment than in the High consumer pressure treatment where the rate of increase of corals was slow (Figure 2b–d). Other experimental work has shown that in the presence of fishes, corals are more likely to have visible bite marks and slower growth than when fishes are absent (Adam et al., 2022; Sotka & Hay, 2009). Our work suggests that predation on corals by fishes can slow coral recovery but that consumers can also determine how abundant corals are relative to their algal competitors.

Our experiment provides strong evidence that the presence of consumers favors communities with high abundances of corals relative to their algal competitors (Figure 6). At High and Medium levels of consumer pressure, herbivory and corallivory resulted in slow increases in the abundance of corals while limiting the abundance of macroalgae (Figure 2), which favored communities that had more corals than macroalgae (Figure 6). This pattern is similar to other work showing that consumers increase the importance of deterministic processes in community assembly and promote directional community development (Alberti et al., 2017). By contrast, in the Low consumer pressure treatment, all replicates had more algae than corals after 1 year and remained dominated by macroalgae through the end of the experiment (Figure 6). When consumer pressure is greatly reduced, algae are released from top‐down control and can proliferate and negatively affect corals by inhibiting coral settlement or killing recruits (Birrell et al., 2008; Kuffner et al., 2006; Webster et al., 2015), reducing coral growth through competition (Lirman, 2001; Tanner, 1997), and making corals more susceptible to disease (Zaneveld et al., 2016). These negative interactions between corals and algae are well documented, and may be particularly strong early in community development when corals are small (Ferrari et al., 2012), but the degree to which they limit or slow coral recovery after disturbances is not well understood. Our experiment shows that consumers promote the development of communities with more corals than algae, but that communities dominated by corals with relatively little macroalgae can sometimes, but not always, develop when consumer pressure is greatly reduced.

At the lowest level of consumer pressure (Very Low treatment), communities existed along a gradient of abundant coral to abundant macroalgae, indicating that extreme reductions in consumer pressure can lead to surprising community trajectories. At Very Low consumer pressure, coral predators (e.g., butterflyfishes, pufferfishes, filefishes, excavating parrotfishes) were also excluded from our experimental plots in addition to most herbivores, resulting in minimal corallivory and herbivory. Corallivory can slow coral growth and cause mortality (Bulleri et al., 2013; Lenihan & Edmunds, 2010), and in the absence of corallivory, small corals that recruited early in the experiment likely had a competitive edge against macroalgae, enabling them to monopolize space and suppress macroalgae. The fact that corals became abundant in some of the replicates where both corallivores and herbivores were absent suggests that corallivores can negatively impact recovering coral communities. Our findings also agree with other experimental work showing that priority effects can determine the outcomes of coral–algal competition under reduced consumer pressure (Adam et al., 2022).

We report that consumers have strong effects on the relative abundance of common taxa, which likely has consequences for ecosystem processes such as primary production and calcification. For example, community composition can have strong effects on community metabolism, primary production, and decomposition across aquatic and terrestrial systems (Barnas et al., 2025; Bradford et al., 2002; Fields & Silbiger, 2022; Hooper & Vitousek, 1997). Although many have shown that the composition of communities can dictate ecosystem processes, it is unclear how the variability of communities (i.e., beta diversity) potentially translates to variability in ecosystem processes (Mori et al., 2018). This relationship may be particularly consequential on coral reefs where some primary producers are calcifying organisms that contribute to reef accretion (e.g., corals and coralline algae) whereas others (e.g., macroalgae and turf algae) do not. Thus, altered patterns of beta diversity that reflect changes in the relative abundance of benthic organisms with different functional roles may have consequences for the overall rates and variability of ecosystem processes.

Altered patterns of beta diversity under anthropogenic stressors may also have consequences for conservation and ecosystem management. One prevailing concern is that human influences may be causing widespread declines in beta diversity, resulting in biological homogenization (Socolar et al., 2016). However, the main finding from our work, increased beta diversity under decreased consumer pressure, shows the opposite pattern where high between‐site dissimilarity results from the loss of consumers. This suggests that reducing consumer pressure, such as through overfishing, makes the recovery trajectories of reefs more unpredictable following disturbances. Multiple stressors, such as overfishing, nutrient enrichment, and the cyclone that we mimic in our experiment, may be more likely to result in ecological surprises where communities transition to novel species assemblages or ecosystem states (Filbee‐Dexter et al., 2017; Paine et al., 1998). On coral reefs, recovery of corals may be likely following a single disturbance such as a cyclone (Adam et al., 2011; Speare et al., 2025), but this recovery may become more variable when reefs are subjected to disturbances against a backdrop of other stressors such as overfishing or nutrient pollution. Increasingly variable community trajectories may be undesirable from a management perspective if some reefs recover to a coral‐dominated state and others become dominated by algae under similar environmental conditions. These types of ecological surprises resulting from unpredictable community dynamics will make ecosystem management challenging in the face of anthropogenic alterations to top‐down and bottom‐up processes.

## CONFLICT OF INTEREST STATEMENT

The authors declare no conflicts of interest.

## Supporting information


Appendix S1:

## Data Availability

Data (Moorea Coral Reef LTER et al., 2026) are available in the Environmental Data Initiative repository at https://doi.org/10.6073/pasta/fa1511a909fae9b55a5d8a5ccbfa1f60. Code (Speare, 2026) is available in Zenodo at https://doi.org/10.5281/zenodo.20452849.
