# Supplementary material for: Reducing consumer pressure increases community dissimilarity leading to coral‐ or algal‐dominance on a coral reef
Source: Ecology. 2026 Sep 15;107(9):e70507. doi: 10.1002/ecy.70507 (PMC13577821; doi:10.1002/ecy.70507)
Supplement: Supplementary file 1 [file ECY-107-e70507-s001.pdf]

Appendix S1

Reducing consumer pressure increases community dissimilarity leading to coral- or algal-dominance on a coral reef

Kelly E. Speare, Thomas C. Adam, Allison Aplin, Noe Castañeda, Lauren N. Enright, Hannah E. Epstein, Jordan P. Gallagher, Adam Goodman, Olivia Isbell, Christian John, Mackenzie A. Kawahara, Grace Klinges, Mark C. Ladd, Jamie M. McDevitt-Irwin, Rowan H. McLachlan, Nury Molina, Joseph R. Peters, Julianna J. Renzi, Andrew A. Shantz, Andrew R. Thurber, Alex D. Vompe, Rebecca Vega Thurber, Deron E. Burkepile

Ecology

Additional Methods

Effects of the consumer exclusion treatment

In our experiment, we quantified the abundance of herbivorous and corallivorous fishes using timed *in situ* surveys. Surveys were conducted on each exclosure on eight occasions between 2018 and 2022. We recorded the size and species of all herbivorous and corallivorous fishes that were present or entered an exclosure within a five-minute survey. Divers inspected each exclosure for any cryptic fish during the last minute of the survey. Fish lengths were converted to biomass for each individual using allometric scaling relationships from the literature (Moorea Coral Reef LTER and Brooks 2024). Individual fish biomass was then summed for a community biomass for each exclosure. Data from each replicate exclosure at each timepoint were averaged to calculate the mean biomass of each functional group of fishes in each treatment. A list of fish species in each functional group is given in Appendix S1: Table S2.

To analyze data on the abundance of fishes across treatments we first summed the total biomass of herbivores (all functional groups) and corallivores in each exclosure during each sampling point. We then analyzed biomass data for herbivores and corallivores separately. We used mixed effects models with fixed effects of consumer pressure, nutrients, and their interaction, and plot identity was included as a random effect. Biomass data was log transformed (+1) to homogenize variances. Models were constructed using the *lme4* package (Bates et al. 2015) and χ^2 tests were performed with Type II Sums of Squares using the using *anova* from the *car* package (Fox and Weisberg 2018). Model residual diagnostics were evaluated using the *DHARMa* package (Hartig 2024).

Corallivores are low in abundance relative to other consumers (see Figure S2) making it difficult to quantify their abundance using surveys. However, even infrequent bites likely exert strong top-down effects on corals because corallivores remove coral tissue, often excavating parts of the coral skeleton. To evaluate how corallivory varied across levels of consumer pressure in our experiment, we surveyed bites on corals as a proxy for the strength of corallivory. For each coral in the experiment divers searched for bites and recorded whether or not a coral had visible bite marks. Corals were surveyed in November 2021, April 2022, and July 2022. We analyzed data on bite mark prevalence using a glmm with a binomial error structure and a logit link function. The model included consumers, nutrients, and their interaction as fixed effects and coral identity nested within plot identity as a random effect. The model was fit using *glmmTMB* (Brooks et al. 2017) and model diagnostics were evaluated using the *DHARMa* package (Hartig 2024).

Effects of the nutrient enrichment treatment

Divers used 60-ml syringes to slowly draw water from ~3 cm above the benthos in either control or enriched plots. Samples were taken at varying times after the deployment of enrichment diffuser tubes to ensure that enrichment occurred across the duration of diffuser deployment. Immediately after collection, samples were filtered (GF/F) into Falcon tubes, placed on ice, returned to the laboratory and frozen until analyzed. Inorganic nitrogen (nitrite + nitrate) and soluble reactive phosphorus concentrations were determined via autoanalyser at the Marine Science Institute Analytical Laboratory at the University of California, Santa Barbara.

We used linear mixed effects models with fixed effects of nutrient enrichment, days since the diffuser swap (hereafter “time”) and their interaction on the concentration of nitrite + nitrate and phosphate. Sample timepoint was included as a random effect. We also evaluated whether nutrient enrichment had an effect on water nutrient concentrations in samples taken at the end of

the deployment period. Diffuser tubes were typically replaced every 12-16 weeks, so we also analyzed a subset of the data from samples taken after 12 weeks (80 days) of deployment using a linear model with a fixed effect of nutrient enrichment. Models were constructed using the *lme4* package (Bates et al. 2015) and χ^2 tests were performed with Type II Sums of Squares using the using *anova* from the *car* package (Fox and Weisberg 2018). Model residual diagnostics were evaluated using the *DHARMa* package (Hartig 2024).

Testing for interactions between consumers and nutrients on location of group spatial medians

We were interested in the effects of consumer pressure, nutrients, and their interaction on multivariate homogeneity of group dispersions (i.e., beta diversity). However, testing for multivariate homogeneity of dispersions across two factors, such as our experimental design, can be problematic if there is an interaction between two main effects on the location of group spatial medians because differences in the locations of group medians can influence group dispersion(Anderson 2006, Anderson et al. 2006). To evaluate this possibility we took a two step approach (Anderson et al. 2008). First, we calculated group dispersions for a single factor that combined herbivory and nutrient treatments separately for each timepoint (i.e., eight groups representing all unique treatments, Appendix S1: Table S3) using *betadisper* and tested for differences using *anova*. This analysis showed significant differences in group dispersions among groups, which is expected if there are individual or interactive effects of treatments on group dispersions. Second, we tested for individual and interactive effects of treatments (i.e., consumer pressure and nutrients) on the location of group spatial medians (two-way PERMANOVA using *adonis*) for data from each sampling timepoint. This analysis showed no interaction between consumers and nutrients on the location of group medians at any of the timepoints (see Appendix S1: Table S4-S14), ruling out the possibility that an interaction between the two main effects artificially influenced multivariate group dispersions within levels of the main effects.

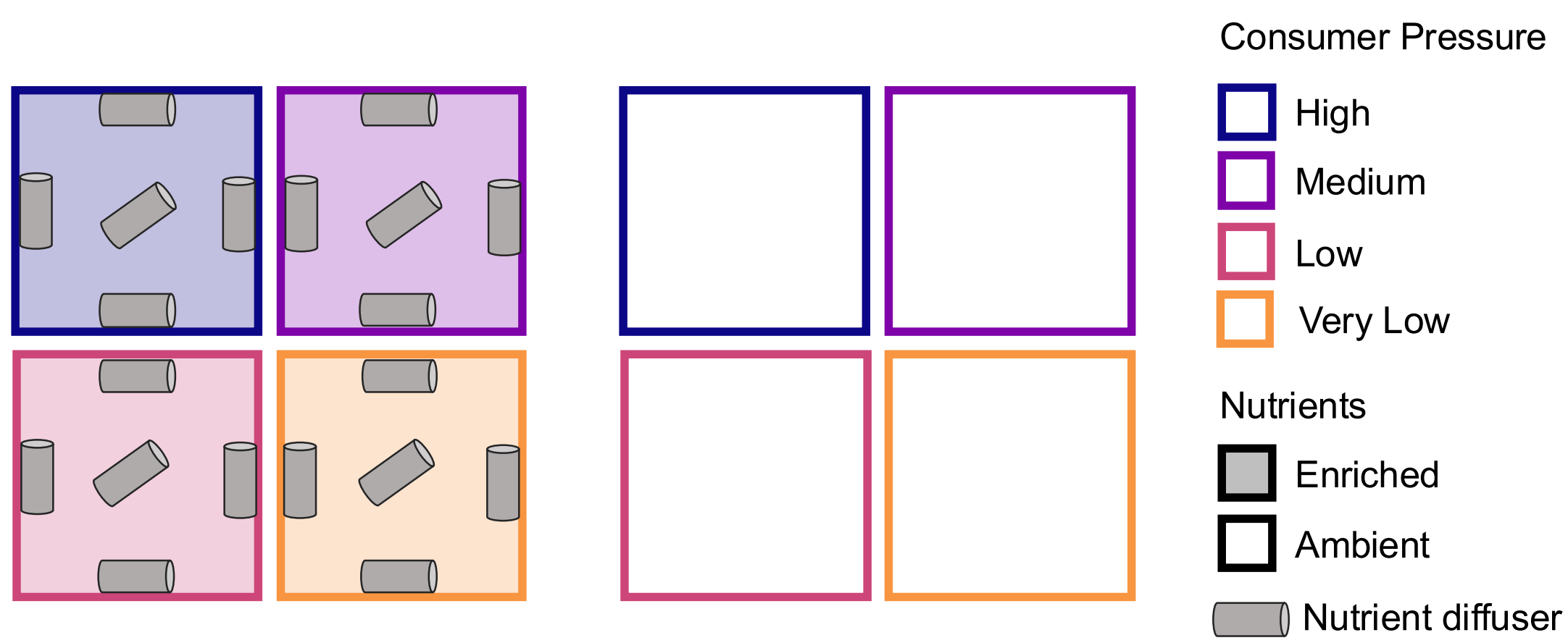

Figure S1. Schematic showing one block of the factorial experiment crossing consumer pressure and nutrients. Each block is replicated four times for n=4 of each combination of treatments.

Additional Results

Effects of the consumer exclusion treatment

Experimental exclosures were highly effective at altering the biomass of herbivorous and corallivorous fishes in our plots. Species of herbivore and corallivore consumers and functional groups to which they belong are listed in Table S1. There was a significant effect of consumer pressure treatment on the biomass of herbivores (χ^2=172.688, df=3, p<0.0001, Figure S2a) where the biomass of herbivores was highest in the High consumer pressure treatment and declined with successively lower levels of consumer pressure. There was not an effect of nutrients on the biomass of herbivorous fishes (χ^2=0.068, df=3, p=0.800) and there was not a significant interaction between consumer pressure and nutrient enrichment (χ^2=0.352, df=3, p=0.950).

For corallivorous fishes, there was a significant effect of consumer pressure treatment on the biomass of corallivores (χ^2=16.520, df=3, p=0.001, Figure S2b) where the biomass of corallivores was highest in the Medium consumer pressure treatment. There was not an effect of nutrients on the biomass of corallivorous fishes (χ^2=0.522, df=1, p=0.470) and there was not a significant interaction between consumer pressure and nutrient enrichment (χ^2=6.359, df=3, p=0.095). Most of the corallivores that visited plots during the timed surveys were butterflyfishes. These abundant and relatively small fishes feed on corals by removing only the coral polyp tissue. Large, excavating corallivores such as filefishes and pufferfishes also feed on corals at this site, but they are less abundant and likely not captured in our 5-minute surveys. These excavating corallivores remove coral tissue and parts of the skeleton and likely have meaningful impacts on coral growth and survivorship, particularly in the High and Medium consumer pressure treatments. However, due to their rarity, we were not able to document differences in their abundance across treatments.

Consumer pressure had a strong effect on the probability that corals had visible bite marks (χ^2=205.37, df=3, p<0.0001), where corals were more likely to be bitten at higher levels of consumer pressure. There was no effect of nutrients (χ^2=0.64, df=1, p=0.424), or interaction between nutrients and consumers (χ^2=1.88, df=3, p=0.600), on the probability that corals had bite marks.

Table S1. Taxa included in benthic groups in Figure 3.

**Taxon**	**Benthic group**
Acanthastrea spp.	corals
Acropora spp.	corals
Amphiroa spp.	macroalgae
Anemone	other
Asparagopsis	macroalgae
Caulerpa spp.	macroalgae
CCA	other
Chlorodesmis	macroalgae
Colpomenia	macroalgae
Cyanobacteria	macroalgae
Dictyota spp.	macroalgae
Foliose Lobophora	macroalgae
Fungiid	corals

Galaxaura spp.	macroalgae
Gibsmithia	macroalgae
Grateloupia	macroalgae
Halimeda spp.	corals
Hydroclathrus	macroalgae
Hypnea	macroalgae
Jania spp.	corals
Leptastrea	corals
Leptoseris	corals
Lobophyla	corals
Long turf	other
Millepora	corals
Montipora spp.	corals
Padina	macroalgae
Pavona	corals
Phymastraea	corals
Pocillopora spp.	corals
Porites spp.	corals
Psammocora	corals
Rubble	other
Sand	other
Sargassum	macroalgae
Soft coral	other
Spatoglossum	macroalgae
Sponge	other
Tridacna	other
Turbinaria	macroalgae
Turf Bare Encrusting Algae	colonizable space
Unknown encrusting coral	corals

Table S2. Functional groups and species of herbivore and corallivore consumers that visited experimental plots.

	**Functional Group**	**Species**
Herbivore	Browser	*Calotomus carolinus*
Herbivore	Browser	*Leptoscarus vaigiensis*
Herbivore	Browser	*Naso lituratus*
Herbivore	Grazer	*Acanthurus achilles*
Herbivore	Grazer	*Acanthurus nigricans*
Herbivore	Grazer	*Acanthurus nigrofuscus*
Herbivore	Grazer	*Acanthurus nigros*
Herbivore	Grazer	*Acanthurus pyroferus*
Herbivore	Grazer	*Zebrasoma scopas*
Herbivore	Grazer	*Zebrasoma* sp.
Only Herbivore	Scraper/Excavator	*Chlorurus spilurus*
Herbivore	Scraper/Excavator	*Scarus forsteni*
Herbivore	Scraper/Excavator	*Scarus frenatus*
Herbivore	Scraper/Excavator	*Scarus globiceps*
Herbivore	Scraper/Excavator	*Scarus niger*
Herbivore	Scraper/Excavator	*Scarus oviceps*
Herbivore	Scraper/Excavator	*Scarus psittacus*
Herbivore	Scraper/Excavator	*Scarus rubroviolaceus*
Corallivore	Corallivore	*Canthigaster* sp.
Corallivore	Corallivore	*Chaetodon lunulatus*
Corallivore	Corallivore	*Chaetodon ornatissimus*
Corallivore	Corallivore	*Chaetodon pelewensis*
Corallivore	Corallivore	*Chaetodon reticulatus*
Corallivore	Corallivore	*Exallias brevis*

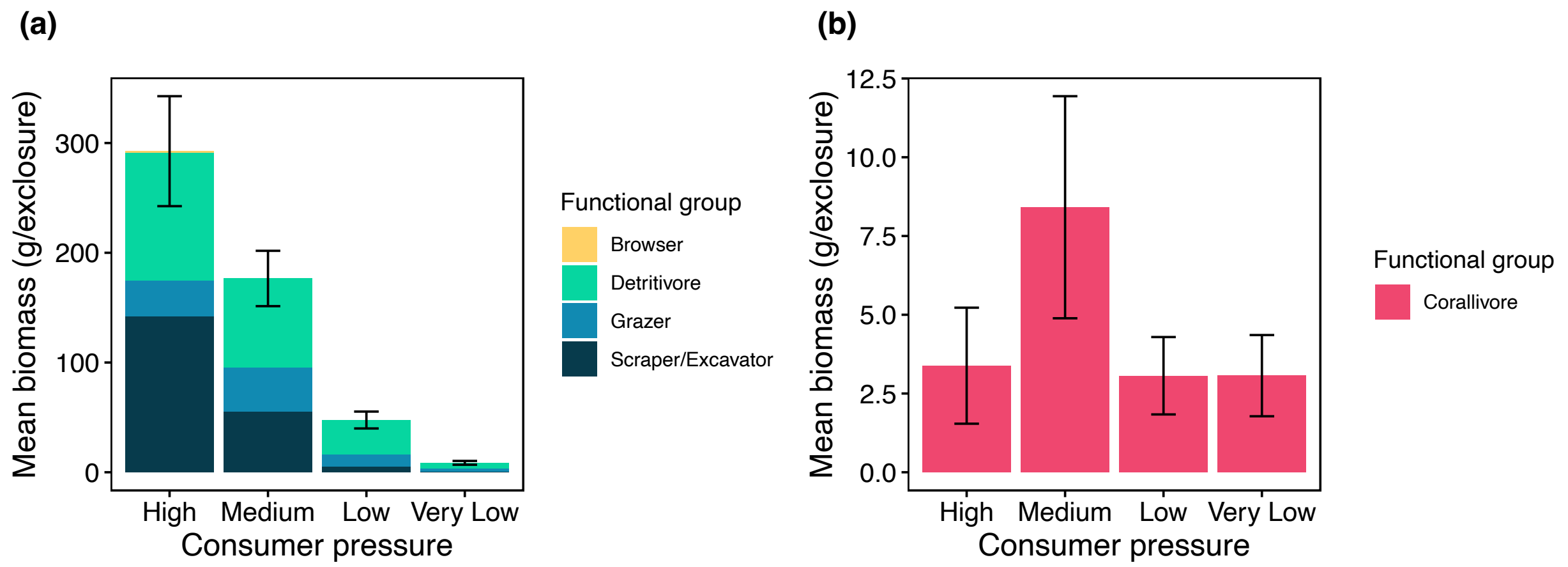

Figure S2. Mean biomass of functional groups of (a) herbivorous and (b) corallivorous fishes in experimental plots observed during 5-minute visual surveys. Error bars represent standard errors for aggregated functional group totals (i.e., all herbivores, all corallivores)

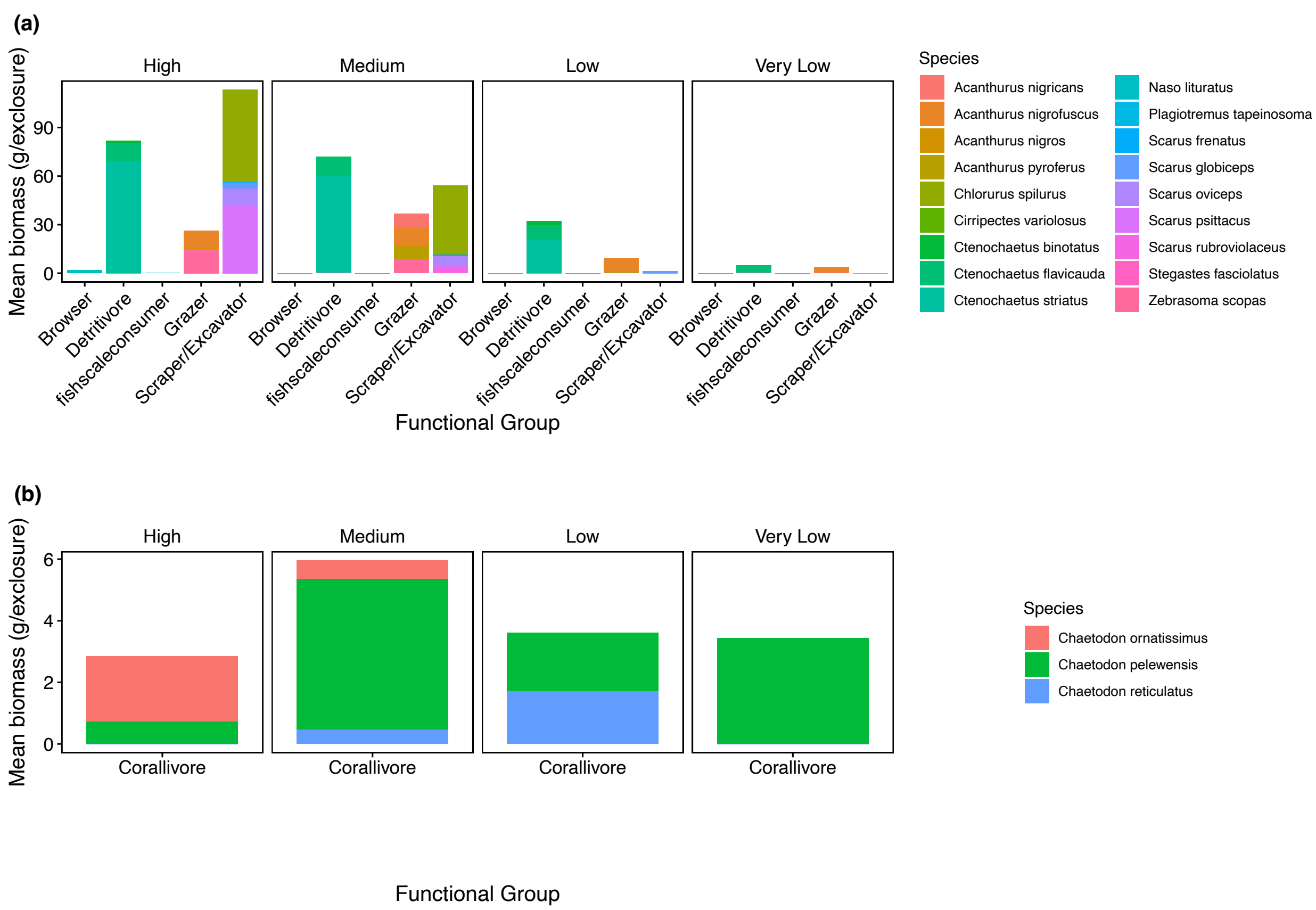

Figure S3. Mean biomass of species within functional groups of (a) herbivorous and (b) corallivorous fishes in experimental plots observed during 5-minute visual surveys. Colors represent different species of fishes.

Effects of the nutrient enrichment treatment

The nutrient enrichment treatment significantly increased the concentration of inorganic nutrients in our experimental plots. For nitrite + nitrate there was a significant effect of nutrient enrichment (χ^2=20.961, df=1, p <0.0001), but not an effect of time (χ^2=1.952, df=1, p =0.165). There was a significant interaction between nutrient enrichment and time (χ^2=7.443, df=1, p<0.006) where the effect of enrichment decreased with time. For soluble reactive phosphorus there was a significant effect of nutrient enrichment (χ^2=12.221, df=1, p<0.001), but not an effect of time (χ^2=0.0371, df=1, p=0.543). There was no interaction between nutrient enrichment and time (χ^2=2.270, df=1, p=0.132). The analysis of data from samples taken after >80 days of diffuser deployment showed that nutrient enrichment did not have a significant effect on nitrite + nitrate ($F_{1,29}$=3.79, p=0.0612) or soluble reactive phosphorus ($F_{1,29}$=2.856, df=1, p=0.102).

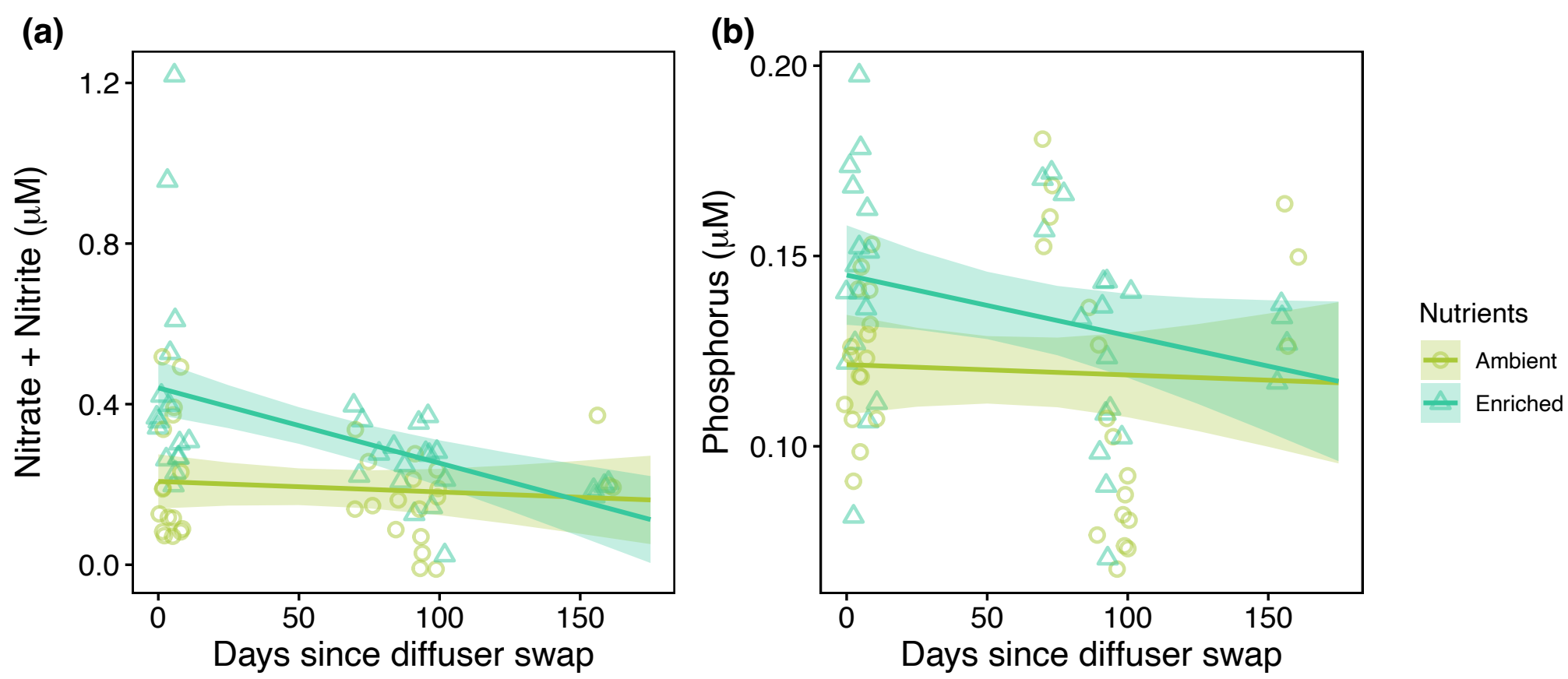

Figure S4. Patterns of (a) nitrate + nitrite and (b) soluble reactive phosphorus concentrations in the water of experimental plots through time. Points represent samples from individual plots. Teal colored triangles represent data from the plots with enriched nutrients and green circles represent data from the plots with ambient nutrients.

Table S3: Results of the test of homogeneity of group dispersions for a single factor that combined levels of consumer pressure and nutrient enrichment (8 groups) for benthic community data

Timepoint		Df	Sum Sq	Mean Sq	F value	Pr(>F)
T0: Jul 2018	Groups	7	0.016	0.0023	0.91	0.5 2
	Residuals	24	0.061	0.0025	NA	NA
T1: Nov 2018	Groups	7	0.014	0.0020	0.57	0.77
	Residuals	24	0.084	0.0035	NA	NA
T2: Mar 2019	Groups	7	0.021	0.0031	1.3	0.23
	Residuals	24	0.057	0.0024	NA	NA
T3: July 2019	Groups	7	0.028	0.0040	1.6	0.12
	Residuals	24	0.061	0.0026	NA	NA
T4: Nov 2019	Groups	7	0.087	0.012	4.0	0.0054
	Residuals	22	0.071	0.0031	NA	NA
T6: Aug 2020	Groups	7	0.034	0.0049	1.2	0.36
	Residuals	24	0.10	0.0043	NA	NA
T8: May 2021	Groups	7	0.15	0.022	3.6	0.0089
	Residuals	24	0.14	0.0061	NA	NA
T9: Jul 2021	Groups	7	0.10	0.014	3.2	0.016
	Residuals	24	0.12	0.0045	NA	NA
T10: Nov 2021	Groups	7	0.076	0.011	3.1	0.020
	Residuals	24	0.087	0.0036	NA	NA
T11: Apr 2022	Groups	7	0.072	0.010	2.7	0.033
	Residuals	24	0.093	0.0039	NA	NA
T12: Jul 2022	Groups	7	0.062	0.0088	1.3	0.27
	Residuals	24	0.16	0.0066	NA	NA

Table S4: Timepoint T0 (July 2018), results from two-way PERMANOVA testing for differences in the location of group centroids

	Df	SumOfSqs	R2	F	Pr(>F)
Consumers	3	0.024	0.064	0.63	0.78
Nutrients	1	0.00047	0.0013	0.037	0.96
Consumers:Nutrients	3	0.044	0.12	1.1	0.36
Residual	24	0.31	0.82	NA	NA
Total	31	0.38	1	NA	NA

Table S5: Timepoint T1 (November 2018)**,** results from two-way PERMANOVA testing for differences in the location of group centroids

	Df	SumOfSqs	R2	F	Pr(>F)
Consumers	3	0.053	0.13	1.4	0.16
Nutrients	1	0.027	0.066	2.2	0.090
Consumers:Nutrients	3	0.033	0.080	0.88	0.53
Residual	24	0.30	0.73	NA	NA
Total	31	0.42	1	NA	NA

Table S6: Timepoint T2 (March 2019), results from two-way PERMANOVA testing for differences in the location of group centroids

	**Df**	**SumOfSqs**	**R2**	**F**	**Pr(>F)**
Consumers	3	0.14	0.22	2.9	0.004
Nutrients	1	0.039	0.064	2.5	0.047
Consumers:Nutrients	3	0.058	0.095	1.2	0.25
Residual	24	0.38	0.62	NA	NA
Total	31	0.61	1	NA	NA

Table S7: Timepoint T3 (July 2019), results from two-way PERMANOVA testing for differences in the location of group centroids

	**Df**	**SumOfSqs**	**R2**	**F**	**Pr(>F)**
Consumers	3	0.44	0.46	8.2	0.0010
Nutrients	1	0.031	0.032	1.7	0.14
Consumers:Nutrients	3	0.062	0.064	1.1	0.31
Residual	24	0.43	0.45	NA	NA
Total	31	0.97	1	NA	NA

Table S8: Timepoint T4 (November 2019), results from two-way PERMANOVA testing for differences in the location of group centroids

	**Df**	**SumOfSqs**	**R2**	**F**	**Pr(>F)**
Consumers	3	0.76	0.49	9.1	0.001
Nutrients	1	0.070	0.045	2.5	0.052
Consumers:Nutrients	3	0.087	0.057	1.0	0.41
Residual	22	0.64	0.41	NA	NA
Total	29	1.6	1	NA	NA

Table S9: Timepoint T6 (August 2020), results from two-way PERMANOVA testing for differences in the location of group centroids

	**Df**	**SumOfSqs**	**R2**	**F**	**Pr(>F)**
Consumers	3	1.1	0.59	14	0.0010
Nutrients	1	0.075	0.042	3.0	0.050
Consumers:Nutrients	3	0.050	0.028	0.66	0.72
Residual	24	0.61	0.34	NA	NA
Total	31	1.8	1	NA	NA

Table S10: Timepoint T8 (May 2021), results from two-way PERMANOVA testing for differences in the location of group centroids

	**Df**	**SumOfSqs**	**R2**	**F**	**Pr(>F)**
Consumers	3	1.5	0.56	13	0.0010
Nutrients	1	0.10	0.038	2.6	0.054
Consumers:Nutrients	3	0.14	0.053	1.2	0.27
Residual	24	0.91	0.35	NA	NA
Total	31	2.6	1	NA	NA

Table S11: Timepoint T9 (July 2021), results from two-way PERMANOVA testing for differences in the location of group centroids

	Df	SumOfSqs	R2	F	Pr(>F)
Consumers	3	1.6	0.58	14	0.0010
Nutrients	1	0.085	0.031	2.1	0.095
Consumers:Nutrients	3	0.12	0.042	0.98	0.43
Residual	24	0.95	0.34	NA	NA
Total	31	2.8	1.0	NA	NA

Table S12: Timepoint T10 (November 2021), results from two-way PERMANOVA testing for differences in the location of group centroids

	Df	SumOfSqs	R2	F	Pr(>F)
Consumers	3	1.1	0.49	11	0.0010
Nutrients	1	0.18	0.084	5.5	0.0030
Consumers:Nutrients	3	0.13	0.059	1.3	0.23
Residual	24	0.80	0.37	NA	NA
Total	31	2.2	1.0	NA	NA

Table S13: Timepoint T11 (April 2022), results from two-way PERMANOVA testing for differences in the location of group centroids

	Df	SumOfSqs	R2	F	Pr(>F)
Consumers	3	1.5	0.53	12	0.0010
Nutrients	1	0.19	0.067	4.6	0.0050
Consumers:Nutrients	3	0.15	0.056	1.3	0.25
Residual	24	0.98	0.35	NA	NA
Total	31	2.8	1.0	NA	NA

Table S14: Timepoint T12 (July 2022), results from two-way PERMANOVA testing for differences in the location of group centroids

	Df	SumOfSqs	R2	F	Pr(>F)
Consumers	3	1.4	0.51	11	0.0010
Nutrients	1	0.15	0.052	3.2	0.020
Consumers:Nutrients	3	0.15	0.053	1.1	0.34
Residual	24	1.1	0.39	NA	NA
Total	31	2.8	1.0	NA	NA

Table S15: Results of similarity percentage analysis contrasting communities in the High and Very Low levels of consumer pressure

Taxa	average	sd	ratio	ava	avb	cumsum	p
Turf algae, bare space, and encrusting algae	0.24	0.06	3.69	20.50	67.56	0.39	0.01
Montipora spp.	0.09	0.07	1.25	21.96	3.30	0.55	0.01
Turbinaria	0.05	0.03	1.74	12.38	2.77	0.63	0.24
Foliose Lobophora	0.04	0.04	1.07	8.02	0.82	0.70	0.03
Halimeda spp.	0.04	0.04	0.89	8.71	2.70	0.76	0.62
Acropora spp.	0.03	0.03	1.18	8.21	2.07	0.81	0.01
Pocillopora spp.	0.02	0.02	1.14	6.85	4.68	0.85	0.23
Rubble	0.02	0.01	1.26	1.86	5.06	0.88	0.02
Unknown encrusting coral	0.01	0.01	1.24	2.21	2.71	0.89	0.08
Pavona	0.01	0.01	0.79	0.06	1.88	0.91	0.01
Porites spp.	0.01	0.01	0.46	1.31	0.07	0.92	0.12
Sargassum	0.01	0.01	0.96	0.32	1.22	0.93	0.86
Sand	0.01	0.00	1.34	1.29	1.26	0.94	0.87
Dictyota spp.	0.00	0.00	1.36	1.69	1.56	0.95	0.33
Phymastraea	0.00	0.01	0.73	0.79	0.30	0.96	0.17
Long turf	0.00	0.00	0.86	0.12	0.69	0.96	0.62
Unknown macroalgae	0.00	0.01	0.46	0.70	0.00	0.97	0.27
Acanthastrea spp.	0.00	0.01	0.37	0.60	0.00	0.97	0.31
Spatoglossum	0.00	0.00	0.88	0.45	0.33	0.98	0.86
Caulerpa spp.	0.00	0.00	1.05	0.25	0.39	0.98	0.99
Padina	0.00	0.00	0.88	0.40	0.00	0.98	0.01
Jania spp.	0.00	0.00	0.53	0.39	0.00	0.99	0.75
Fungiid	0.00	0.00	0.72	0.34	0.13	0.99	0.19
Galaxaura spp.	0.00	0.00	0.64	0.00	0.32	0.99	0.03
Soft coral	0.00	0.00	0.37	0.24	0.00	1.00	0.27
CCA	0.00	0.00	0.88	0.13	0.19	1.00	0.99
Sponge	0.00	0.00	0.77	0.19	0.00	1.00	0.07
Valonia spp.	0.00	0.00	0.37	0.06	0.00	1.00	0.02

Table S16: Results of similarity percentage analysis contrasting communities in the High and Low levels of consumer pressure

Taxa	average	sd	ratio	ava	avb	cumsum	p
Turf algae, bare space, and encrusting algae	0.16	0.07	2.15	35.33	67.56	0.35	0.02
Montipora spp.	0.08	0.04	1.86	18.23	2.77	0.52	0.01
Turbinaria	0.06	0.03	1.73	13.44	2.70	0.65	0.01
Foliose Lobophora	0.03	0.02	1.42	9.81	3.30	0.72	0.94
Halimeda spp.	0.02	0.03	0.85	4.70	0.82	0.77	0.80
Acropora spp.	0.02	0.01	1.22	2.71	5.06	0.81	0.10
Pocillopora spp.	0.02	0.01	1.31	3.90	4.68	0.84	0.87
Rubble	0.01	0.01	1.08	1.71	2.71	0.86	0.14
Unknown encrusting coral	0.01	0.01	1.04	1.91	1.22	0.89	0.17
Pavona	0.01	0.01	0.79	0.06	1.88	0.91	0.01
Porites spp.	0.01	0.00	1.38	2.20	2.07	0.92	1.00
Sargassum	0.01	0.00	1.32	1.02	1.26	0.93	0.90
Sand	0.00	0.00	1.45	1.22	1.56	0.94	0.59
Dictyota spp.	0.00	0.01	0.61	0.81	0.00	0.95	0.27
Phymastraea	0.00	0.00	0.89	0.76	0.33	0.96	0.44
Long turf	0.00	0.00	0.81	0.00	0.69	0.97	0.56
Unknown macroalgae	0.00	0.00	0.81	0.57	0.19	0.97	0.38
Acanthastrea spp.	0.00	0.01	0.50	0.55	0.00	0.98	0.01
Spatoglossum	0.00	0.00	1.14	0.44	0.39	0.99	0.93
Caulerpa spp.	0.00	0.00	0.59	0.20	0.30	0.99	0.79
Padina	0.00	0.00	0.64	0.00	0.32	0.99	0.01
Jania spp.	0.00	0.00	0.78	0.12	0.13	1.00	0.79
Fungiid	0.00	0.00	0.53	0.06	0.07	1.00	0.82
Galaxaura spp.	0.00	0.00	0.37	0.07	0.00	1.00	0.01
Soft coral	0.00	0.00	0.37	0.07	0.00	1.00	0.94
CCA	0.00	0.00	0.37	0.06	0.00	1.00	0.51
Sponge	0.00	0.00	0.37	0.06	0.00	1.00	0.59
Valonia spp.	0.00	0.00	NA	0.00	0.00	1.00	NA
Tridacna	0.00	0.00	NA	0.00	0.00	1.00	NA
Amphiroa spp.	0.00	0.00	NA	0.00	0.00	1.00	NA
Anemone	0.00	0.00	NA	0.00	0.00	1.00	NA
Asparagopsis	0.00	0.00	NA	0.00	0.00	1.00	NA
Chlorodesmis	0.00	0.00	NA	0.00	0.00	1.00	NA
Colpomenia	0.00	0.00	NA	0.00	0.00	1.00	NA
Cyanobacteria	0.00	0.00	NA	0.00	0.00	1.00	NA
Gibsmithia	0.00	0.00	NA	0.00	0.00	1.00	NA
Grateloupia	0.00	0.00	NA	0.00	0.00	1.00	NA
Hydroclathrus	0.00	0.00	NA	0.00	0.00	1.00	NA
Hypnea	0.00	0.00	NA	0.00	0.00	1.00	NA
Leptastrea	0.00	0.00	NA	0.00	0.00	1.00	NA
Leptoseris	0.00	0.00	NA	0.00	0.00	1.00	NA
Lobophyla	0.00	0.00	NA	0.00	0.00	1.00	NA
Millepora	0.00	0.00	NA	0.00	0.00	1.00	NA
Psammocora	0.00	0.00	NA	0.00	0.00	1.00	NA

Table S17: Results of similarity percentage analysis contrasting communities in the High and Medium levels of consumer pressure

Taxa	average	sd	ratio	ava	avb	cumsum	p
Turf algae, bare space, and encrusting algae	0.07	0.05	1.37	53.26	67.56	0.28	1.00
Montipora spp.	0.03	0.03	1.08	9.76	3.30	0.40	0.90
Turbinaria	0.02	0.01	1.54	6.40	2.77	0.49	0.99
Foliose Lobophora	0.02	0.01	1.31	3.98	2.70	0.55	1.00
Halimeda spp.	0.02	0.01	1.40	5.08	4.68	0.61	0.71
Acropora spp.	0.01	0.01	1.13	2.95	5.06	0.67	0.44
Pocillopora spp.	0.01	0.01	1.70	3.14	2.71	0.71	0.05
Rubble	0.01	0.01	0.85	2.45	0.82	0.76	1.00
Unknown encrusting coral	0.01	0.01	1.05	2.09	1.26	0.79	0.15
Pavona	0.01	0.01	0.77	0.19	1.88	0.83	0.01
Porites spp.	0.01	0.01	1.30	3.07	2.07	0.86	0.96
Sargassum	0.01	0.01	1.03	1.15	1.22	0.89	0.64
Sand	0.01	0.01	0.92	1.59	0.39	0.91	0.01
Dictyota spp.	0.01	0.00	1.36	1.99	1.56	0.93	0.15
Phymastraea	0.01	0.00	1.26	1.07	0.69	0.95	0.01
Long turf	0.00	0.00	1.14	0.67	0.33	0.96	0.80
Unknown macroalgae	0.00	0.00	0.94	0.49	0.19	0.97	0.60
Acanthastrea spp.	0.00	0.00	0.50	0.14	0.30	0.98	0.79
Spatoglossum	0.00	0.00	0.64	0.00	0.32	0.99	0.04
Caulerpa spp.	0.00	0.00	0.67	0.07	0.13	0.99	0.75
Padina	0.00	0.00	0.57	0.15	0.00	0.99	0.02
Jania spp.	0.00	0.00	0.57	0.13	0.00	0.99	0.79
Fungiid	0.00	0.00	0.37	0.08	0.00	1.00	0.76
Galaxaura spp.	0.00	0.00	0.37	0.00	0.07	1.00	0.82
Soft coral	0.00	0.00	0.37	0.06	0.00	1.00	0.01
CCA	0.00	0.00	0.37	0.06	0.00	1.00	0.97
Sponge	0.00	0.00	NA	0.00	0.00	1.00	NA
Valonia spp.	0.00	0.00	NA	0.00	0.00	1.00	NA
Tridacna	0.00	0.00	NA	0.00	0.00	1.00	NA
Amphiroa spp.	0.00	0.00	NA	0.00	0.00	1.00	NA
Anemone	0.00	0.00	NA	0.00	0.00	1.00	NA
Asparagopsis	0.00	0.00	NA	0.00	0.00	1.00	NA
Chlorodesmis	0.00	0.00	NA	0.00	0.00	1.00	NA
Colpomenia	0.00	0.00	NA	0.00	0.00	1.00	NA
Cyanobacteria	0.00	0.00	NA	0.00	0.00	1.00	NA
Gibsmithia	0.00	0.00	NA	0.00	0.00	1.00	NA
Grateloupia	0.00	0.00	NA	0.00	0.00	1.00	NA
Hydroclathrus	0.00	0.00	NA	0.00	0.00	1.00	NA
Hypnea	0.00	0.00	NA	0.00	0.00	1.00	NA
Leptastrea	0.00	0.00	NA	0.00	0.00	1.00	NA
Leptoseris	0.00	0.00	NA	0.00	0.00	1.00	NA
Lobophyla	0.00	0.00	NA	0.00	0.00	1.00	NA
Millepora	0.00	0.00	NA	0.00	0.00	1.00	NA
Psammocora	0.00	0.00	NA	0.00	0.00	1.00	NA

Table S18: Results of similarity percentage analysis contrasting communities in the Medium and Low levels of consumer pressure

Taxa	average	sd	ratio	ava	avb	cumsum	p
Turf algae, bare space, and encrusting algae	0.09	0.05	1.82	53.26	35.33	0.26	0.96
Montipora spp.	0.06	0.04	1.44	6.40	18.23	0.42	0.02
Turbinaria	0.05	0.03	1.62	3.98	13.44	0.56	0.01
Foliose Lobophora	0.03	0.02	1.37	9.76	9.81	0.65	0.94
Halimeda spp.	0.02	0.02	0.99	2.45	4.70	0.72	0.77
Acropora spp.	0.02	0.01	1.34	5.08	3.90	0.76	0.91
Pocillopora spp.	0.01	0.01	1.34	2.95	2.71	0.79	0.82
Rubble	0.01	0.01	1.00	1.15	1.91	0.82	0.14
Unknown encrusting coral	0.01	0.01	1.00	2.09	1.02	0.85	0.15
Pavona	0.01	0.01	1.28	3.07	2.20	0.87	0.99
Porites spp.	0.01	0.01	1.48	3.14	1.71	0.90	0.76
Sargassum	0.01	0.01	0.90	1.59	0.44	0.91	0.02
Sand	0.01	0.00	1.14	1.07	0.00	0.93	0.03
Dictyota spp.	0.00	0.00	1.32	1.99	1.22	0.94	0.47
Phymastraea	0.00	0.00	1.10	0.67	0.76	0.95	0.19
Long turf	0.00	0.01	0.64	0.06	0.81	0.96	0.19
Unknown macroalgae	0.00	0.00	1.01	0.49	0.57	0.97	0.06
Acanthastrea spp.	0.00	0.01	0.50	0.00	0.55	0.98	0.05
Spatoglossum	0.00	0.00	0.64	0.14	0.20	0.99	0.92
Caulerpa spp.	0.00	0.00	0.83	0.19	0.06	0.99	1.00
Padina	0.00	0.00	0.67	0.13	0.07	0.99	0.79
Jania spp.	0.00	0.00	0.68	0.07	0.12	0.99	0.89
Fungiid	0.00	0.00	0.57	0.15	0.00	0.99	0.01
Galaxaura spp.	0.00	0.00	0.37	0.08	0.00	1.00	0.76
Soft coral	0.00	0.00	0.37	0.00	0.07	1.00	0.15
CCA	0.00	0.00	0.37	0.06	0.00	1.00	0.01
Sponge	0.00	0.00	0.37	0.00	0.06	1.00	0.68
Valonia spp.	0.00	0.00	0.37	0.00	0.06	1.00	0.95
Tridacna	0.00	0.00	0.37	0.00	0.06	1.00	0.59
Amphiroa spp.	0.00	0.00	NA	0.00	0.00	1.00	NA
Anemone	0.00	0.00	NA	0.00	0.00	1.00	NA
Asparagopsis	0.00	0.00	NA	0.00	0.00	1.00	NA
Chlorodesmis	0.00	0.00	NA	0.00	0.00	1.00	NA
Colpomenia	0.00	0.00	NA	0.00	0.00	1.00	NA
Cyanobacteria	0.00	0.00	NA	0.00	0.00	1.00	NA
Gibsmithia	0.00	0.00	NA	0.00	0.00	1.00	NA
Grateloupia	0.00	0.00	NA	0.00	0.00	1.00	NA
Hydroclathrus	0.00	0.00	NA	0.00	0.00	1.00	NA
Hypnea	0.00	0.00	NA	0.00	0.00	1.00	NA
Leptastrea	0.00	0.00	NA	0.00	0.00	1.00	NA
Leptoseris	0.00	0.00	NA	0.00	0.00	1.00	NA
Lobophyla	0.00	0.00	NA	0.00	0.00	1.00	NA
Millepora	0.00	0.00	NA	0.00	0.00	1.00	NA
Psammocora	0.00	0.00	NA	0.00	0.00	1.00	NA

Table S19: Results of similarity percentage analysis contrasting communities in the Low and Very Low levels of consumer pressure

Taxa	average	sd	ratio	ava	avb	cumsum	p
Turf algae, bare space, and encrusting algae	0.08	0.06	1.39	20.50	35.33	0.19	1.00
Montipora spp.	0.07	0.07	1.04	21.96	9.81	0.36	0.06
Turbinaria	0.05	0.03	1.54	8.71	13.44	0.48	0.07
Foliose Lobophora	0.05	0.04	1.31	12.38	18.23	0.59	0.47
Halimeda spp.	0.04	0.03	1.23	8.02	4.70	0.68	0.03
Acropora spp.	0.03	0.03	1.18	8.21	2.20	0.76	0.01
Pocillopora spp.	0.02	0.02	1.06	6.85	3.90	0.81	0.24
Rubble	0.01	0.01	1.20	1.86	2.71	0.84	0.81
Unknown encrusting coral	0.01	0.01	0.86	0.32	1.91	0.86	0.34
Pavona	0.01	0.01	1.29	2.21	1.71	0.88	0.86
Porites spp.	0.01	0.01	0.46	1.31	0.06	0.89	0.08
Sargassum	0.01	0.00	1.35	1.29	1.02	0.91	0.79
Sand	0.00	0.01	0.78	0.39	0.81	0.92	0.05
Dictyota spp.	0.00	0.01	0.74	0.79	0.20	0.93	0.16
Phymastraea	0.00	0.00	0.92	0.45	0.76	0.94	0.32
Long turf	0.00	0.00	1.32	1.69	1.22	0.95	0.85
Unknown macroalgae	0.00	0.01	0.49	0.70	0.07	0.95	0.15
Acanthastrea spp.	0.00	0.01	0.41	0.60	0.06	0.96	0.11
Spatoglossum	0.00	0.00	0.78	0.13	0.57	0.97	0.39
Caulerpa spp.	0.00	0.01	0.50	0.00	0.55	0.98	0.02
Padina	0.00	0.00	1.02	0.25	0.44	0.98	0.99
Jania spp.	0.00	0.00	0.88	0.40	0.00	0.99	0.01
Fungiid	0.00	0.00	0.72	0.34	0.12	0.99	0.22
Galaxaura spp.	0.00	0.00	0.46	0.24	0.06	0.99	0.14
Soft coral	0.00	0.00	0.77	0.19	0.00	1.00	0.10
CCA	0.00	0.00	0.37	0.12	0.00	1.00	1.00
Sponge	0.00	0.00	0.52	0.06	0.06	1.00	0.99
Valonia spp.	0.00	0.00	0.37	0.00	0.07	1.00	0.14
Tridacna	0.00	0.00	0.37	0.06	0.00	1.00	0.01
Amphiroa spp.	0.00	0.00	NA	0.00	0.00	1.00	NA
Anemone	0.00	0.00	NA	0.00	0.00	1.00	NA
Asparagopsis	0.00	0.00	NA	0.00	0.00	1.00	NA
Chlorodesmis	0.00	0.00	NA	0.00	0.00	1.00	NA
Colpomenia	0.00	0.00	NA	0.00	0.00	1.00	NA
Cyanobacteria	0.00	0.00	NA	0.00	0.00	1.00	NA
Gibsmithia	0.00	0.00	NA	0.00	0.00	1.00	NA
Grateloupia	0.00	0.00	NA	0.00	0.00	1.00	NA
Hydroclathrus	0.00	0.00	NA	0.00	0.00	1.00	NA
Hypnea	0.00	0.00	NA	0.00	0.00	1.00	NA
Leptastrea	0.00	0.00	NA	0.00	0.00	1.00	NA
Leptoseris	0.00	0.00	NA	0.00	0.00	1.00	NA
Lobophyla	0.00	0.00	NA	0.00	0.00	1.00	NA
Millepora	0.00	0.00	NA	0.00	0.00	1.00	NA
Psammocora	0.00	0.00	NA	0.00	0.00	1.00	NA

Table S20: Results of similarity percentage analysis contrasting communities in the Ambient and Enriched levels of nutrients

Taxa	average	sd	ratio	ava	avb	cumsum	p
Turf algae, bare space, and encrusting algae	0.11	0.08	1.36	47.12	41.21	0.28	0.66
Montipora spp.	0.05	0.06	0.93	8.10	14.32	0.42	0.17
Foliose Lobophora	0.05	0.04	1.23	12.67	7.22	0.53	0.13
Halimeda spp.	0.04	0.04	1.12	4.73	9.68	0.63	0.05
Turbinaria	0.03	0.03	0.98	5.54	2.46	0.70	0.12
Pocillopora spp.	0.02	0.02	1.19	4.13	6.12	0.75	0.10
Acropora spp.	0.02	0.02	0.89	3.49	4.29	0.80	0.26
Rubble	0.01	0.01	1.19	2.77	3.52	0.83	0.49
Pavona	0.01	0.01	1.21	2.36	2.52	0.85	0.88
Sand	0.01	0.01	0.91	1.25	1.05	0.87	0.90
Unknown encrusting coral	0.01	0.01	1.04	1.27	1.56	0.89	0.67
CCA	0.00	0.01	0.52	0.39	0.71	0.90	0.91
Porites spp.	0.00	0.01	0.78	0.41	0.92	0.92	0.11
Phymastraea	0.00	0.00	1.35	1.64	1.59	0.93	0.79
Dictyota spp.	0.00	0.00	0.86	0.58	0.36	0.94	0.51
Sargassum	0.00	0.01	0.33	0.69	0.03	0.94	0.26
Acanthastrea spp.	0.00	0.00	0.98	0.53	0.57	0.95	0.94
Padina	0.00	0.01	0.59	0.63	0.00	0.96	0.02
Long turf	0.00	0.01	0.61	0.25	0.47	0.97	0.59
Caulerpa spp.	0.00	0.00	0.83	0.45	0.24	0.97	0.41
Unknown macroalgae	0.00	0.01	0.40	0.16	0.29	0.98	0.29
Spatoglossum	0.00	0.01	0.29	0.33	0.00	0.98	0.06
Fungiid	0.00	0.00	0.66	0.16	0.17	0.99	0.57
Cyanobacteria	0.00	0.00	0.33	0.06	0.21	0.99	0.78
Jania spp.	0.00	0.00	0.51	0.06	0.14	0.99	0.44
Asparagopsis	0.00	0.00	0.40	0.03	0.13	0.99	0.42
Galaxaura spp.	0.00	0.00	0.32	0.12	0.03	1.00	0.46
Soft_coral	0.00	0.00	0.54	0.03	0.10	1.00	0.10
Sponge	0.00	0.00	0.36	0.04	0.04	1.00	0.57
Lobophyla	0.00	0.00	0.26	0.00	0.04	1.00	0.01
Valonia spp.	0.00	0.00	0.26	0.00	0.03	1.00	0.01
Tridacna	0.00	0.00	0.26	0.03	0.00	1.00	0.01
Amphiroa spp.	0.00	0.00	NA	0.00	0.00	1.00	NA
Anemone	0.00	0.00	NA	0.00	0.00	1.00	NA
Chlorodesmis	0.00	0.00	NA	0.00	0.00	1.00	NA
Colpomenia	0.00	0.00	NA	0.00	0.00	1.00	NA
Gibsmithia	0.00	0.00	NA	0.00	0.00	1.00	NA
Grateloupia	0.00	0.00	NA	0.00	0.00	1.00	NA
Hydroclathrus	0.00	0.00	NA	0.00	0.00	1.00	NA
Hypnea	0.00	0.00	NA	0.00	0.00	1.00	NA
Leptastrea	0.00	0.00	NA	0.00	0.00	1.00	NA
Leptoseris	0.00	0.00	NA	0.00	0.00	1.00	NA
Millepora	0.00	0.00	NA	0.00	0.00	1.00	NA
Psammocora	0.00	0.00	NA	0.00	0.00	1.00	NA

References

Anderson, M. J. 2006. Distance-based tests for homogeneity of multivariate dispersions. Biometrics 62:245–253.

Anderson, M. J., K. E. Ellingsen, and B. H. McArdle. 2006. Multivariate dispersion as a measure of beta diversity. Ecology Letters 9:683–693.

Anderson, M. J., R. N. Gorley, K. S. Clarke, M. Anderson, R. N. Gorley, K. Clarke, and M. Andersom. 2008. PERMANOVA+ for PRIMER. Guide to software and statistical methods. PRIMER-E, Plymouth, UK.

Brooks, M., K. Kristensen, K. van Benthem, A. Magnusson, C. Berg, A. Nielsen, H. Skaug, M. Mächler, and B. Bolker. 2017. GlmmTMB balances speed and flexibility among packages for zero-inflated generalized linear mixed modeling. The R journal 9:378.

Fox, J., and S. Weisberg. 2018. An R companion to applied regression. Third edition. SAGE Publications, Thousand Oaks, CA.

Hartig, F. 2024. DHARMa: Residual diagnostics for hierarchical (multi-level / mixed) regression models. The R Foundation.

Moorea Coral Reef LTER, and A. Brooks. 2024, September 27. MCR LTER: Coral Reef: Long-term Population and Community Dynamics: Fishes, ongoing since 2005.
